# Synthesis of a highly thermostable insulin by phenylalanine conjugation at B29 Lysine

**DOI:** 10.1038/s42004-024-01241-z

**Published:** 2024-07-23

**Authors:** Shantanu Sen, Rafat Ali, Akanksha Onkar, Shivani Verma, Quazi Taushif Ahmad, Pratibha Bhadauriya, Pradip Sinha, Nisanth N. Nair, Subramaniam Ganesh, Sandeep Verma

**Affiliations:** 1 Department of Chemistry, Indian Institute of Technology Kanpur, Kanpur, 208016 UP India; 2 Department of Biological Sciences & Bioengineering, Indian Institute of Technology Kanpur, Kanpur, 208016 UP India; 3 Mehta Family Centre for Engineering in Medicine, Indian Institute of Technology, Kanpur, 208016 UP India; 4 Gangwal School of Medical Sciences and Technology, Indian Institute of Technology, Kanpur, 208016 UP India; 5 Present Address: Department of Laboratory Medicine, University of California San Francisco, San Francisco, 94143 CA USA

**Keywords:** Peptides, Protein aggregation, Chemical modification, Peptides, Protein aggregation

## Abstract

Globally, millions of diabetic patients require daily life-saving insulin injections. Insulin heat-lability and fibrillation pose significant challenges, especially in parts of the world without ready access to uninterrupted refrigeration. Here, we have synthesized four human insulin analogs by conjugating ε-amine of B29 lysine of insulin with acetic acid, phenylacetic acid, alanine, and phenylalanine residues. Of these, phenylalanine-conjugated insulin, termed FHI, was the most stable under high temperature (65 °C), elevated salt stress (25 mM NaCl), and varying pH levels (ranging from highly acidic pH 1.6 to physiological pH 7.4). It resists fibrillation for a significantly longer duration with sustained biological activity in in vitro, ex vivo, and in vivo and displays prolonged stability over its native counterpart. We further unravel the critical interactions, such as additional aromatic π-π interactions and hydrogen bonding in FHI, that are notably absent in native insulin. These interactions confer enhanced structural stability of FHI and offer a promising solution to the challenges associated with insulin heat sensitivity.

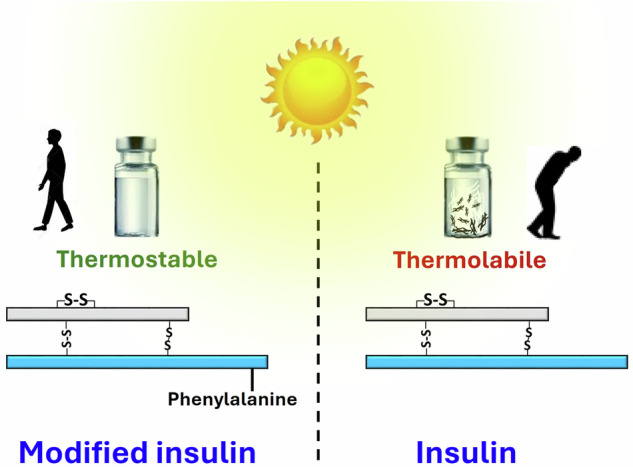

## Abstract (web summary)

Heat-lability and fibrillation of insulin pose significant challenges for insulin storage. Here, the authors report chemically modified analogs of insulin by functionalizing the ε-amine group of B29 Lys with phenylalanine, to improve insulin thermostability and resist fibrillation while maintaining robust in vivo activity.

## Introduction

The discovery of insulin by Banting and Best in 1921 brought high hopes for diabetic patients, and the world is celebrating the centenary of the discovery of this vital hormone^1,2^. Globally, millions of diabetic patients require daily life-saving insulin injections^3^. At high temperatures, insulin degrades, marked by physical degradation, fibrillation, and chemical degradation. The latter is characterized by re-shuffling of disulfide bonds, covalent aggregation, oxidation, de-amidation, formation of covalent polymers, formation of iso-aspartic acid, etc^4–6^. Insulin fibrillation, meaning its amyloid-like aggregation, particularly at high temperatures poses a significant manufacturing, transportation, and storage challenge^7–9^. Injection of degraded insulin often causes amyloidosis and adverse immune responses^5,10–12^.

Previous efforts to mitigate the challenge of insulin fibrillation include the addition of excipients such as aromatic compounds^13,14^, peptides and hybrids^15–19^, nanoparticles^20^, trehalose^21,22^, polyphenols^23^, dyes^24^, metal complexes^25,26^, and hydrophobic interfaces^27^. However, excipients tend to introduce allergic reactions^28,29^. Alternative approaches, which do not entail the use of excipients such as amino acid substitutions^30,31^, incorporation of hydroxy proline^32^, halogenated insulin analogs^33,34^, introduction and replacement of disulfide bonds^35,36^, single chain insulin analog^37–40^, glycosylated insulin analog^41^, and analogs of insulin-like venom of marine snails^42^ have also been explored. However, their syntheses and scale-ups are often time-consuming, involving multiple steps, tedious purifications, and low yields that curtail practical utility.

Synthetic strategies for modification at B29 lysine of insulin were well documented in the literature^43–45^. B29 lysine modification is known to significantly modulate its physio-chemical attributes while preserving the overall three-dimensional structure of insulin. Degludec, Lispro, Detemir, and Glulisine are exemplars of B29 lysine-modified commercially available injectable insulin analogs^46^. The importance of B29 Lys residue is further underscored by the fact that deletion of only one methylene group in its side chain, as in the case of B29Orn-insulin, changes the fibrillation lag time compared to native insulin^31,47^. Several MD studies have also shown enhanced stability of insulin upon conjugation with polyethylene glycol (PEG)^48,49^. While B29 Lys modification is recognized for its impact on the biological properties and insulin fibrillation, its potential to enhance insulin thermostability requires further exploration. Herein, we report the synthesis of four insulin analogs (AcHI, PhacHI, AHI, and FHI) by either masking the ε-amino group of B29 Lys with acetic and phenylacetic acid or by conjugating it with amino acids alanine and phenylalanine. We chose these modifications to investigate their effects on insulin thermostability by functionalizing ε-amine group of B29 Lys. Chemical modification was achieved by either using aliphatic acetyl group to afford AcHI or by employing aromatic phenylacetyl group to afford PhacHI analog. We also used two natural amino acids for functionalization: either alanine to generate AHI or phenylalanine to afford FHI, followed by detailed investigations concerning their effects on insulin thermostability. We obtained high thermostability and resistance to fibrillation in a high-yielding phenylalanine-conjugated insulin derivative, which displayed robust in vivo activity. Our study presents a simple one-step modification to produce a thermostable human insulin variant.

## Results

### Synthesis of insulin analogs

**Fig. 1:**
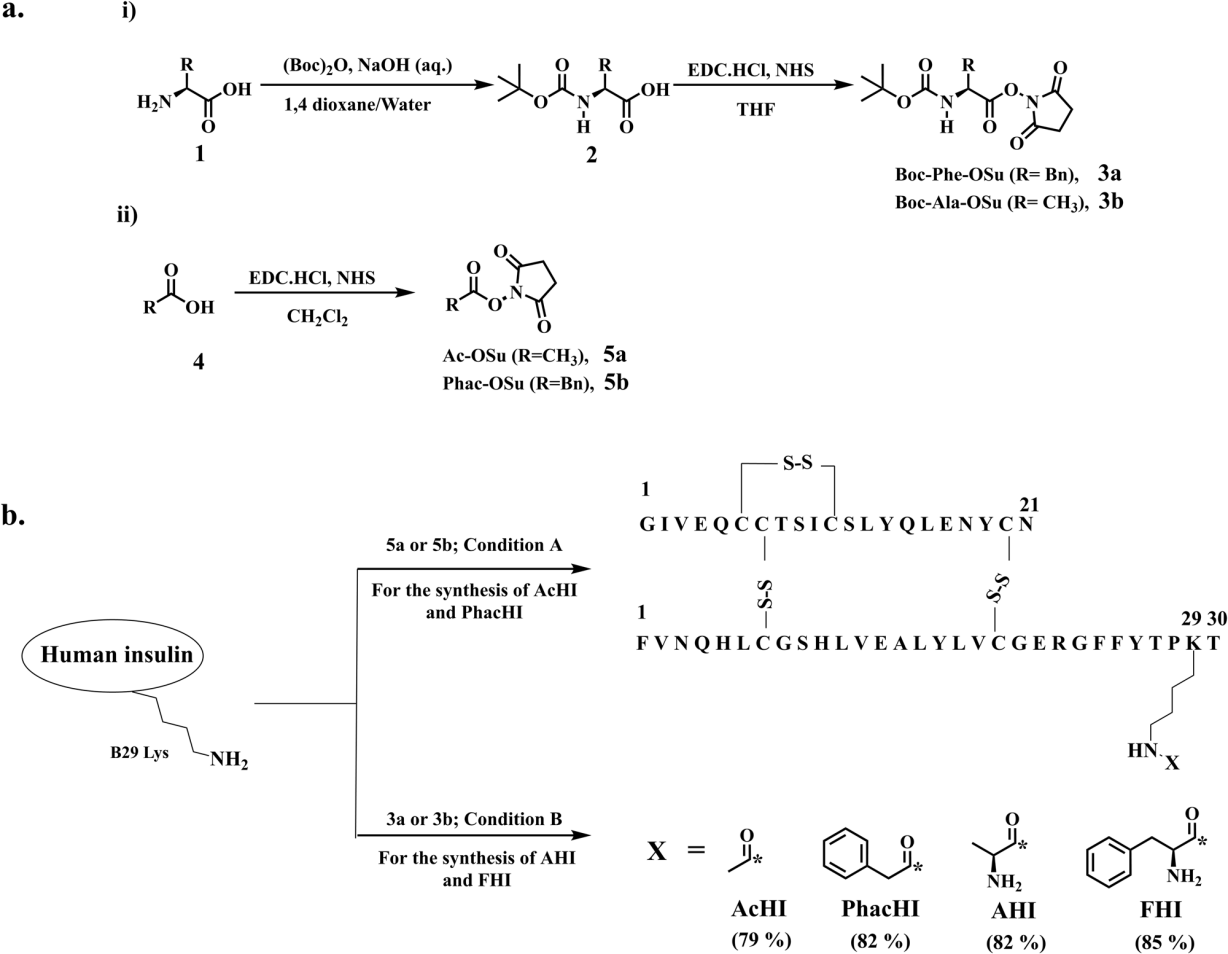
General synthetic scheme and structure of insulin analogs AcHI, PhacHI, AHI, and FHI. **a** General synthetic scheme for the synthesis of active esters. **b** General scheme for the synthesis of newer insulin analogs. Condition A: Na_2_CO_3_/NaHCO_3_ buffer of pH10. Condition B: (1) Na_2_CO_3_/NaHCO_3_ buffer of pH10; (2) Trifluoroacetic acid. The asterisk denotes the point of attachment of carbon to the side chain amino group B29 Lys. Isolated yield of insulin analogs was mentioned in parentheses.

The new insulin analogs are synthesized by conjugating the side chain amine of B29 Lys with the carboxyl-terminal of introduced moieties through amide or isopeptide linkage (Fig. 1b & Scheme S1). The difference in pKa values of *N*-terminal amino groups and an ε-amino group of B29 Lys of insulin allows selective B29 Lys coupling under particular alkaline conditions^50^.

For the synthesis of insulin analogs in which human insulin (HI) conjugated with Acetyl (AcHI), Phenylacetyl (PhacHI), Alanyl (AHI), and Phenylalanyl (FHI) moieties, initially succinimidyl ester of acetic acid, phenylacetic acid, Boc-Alanine and Boc-Phenylalanine were synthesized (Fig. 1a). These succinimidyl esters were then selectively conjugated with the ε-amino group of B29 Lys of insulin (Fig. 1b, Scheme S1). The conjugation reaction was optimized in different sets of sodium carbonate/sodium bicarbonate buffers of pH 9.2, pH 10, and pH 10.6, and individual reactions were monitored by analytical reverse phase HPLC. Of these, pH 10 buffer shows the best outcome regarding efficient conjugation with minimal by-product formation (Fig. S1). On completion of the reaction, crude reaction mixtures were purified by preparative reverse phase HPLC to give AcHI, PhacHI, Boc-AHI, and Boc-FHI as white powder. Boc-AHI and Boc-FHI were treated with trifluoroacetic acid for Boc deprotection to give the desired products, AHI and FHI, respectively. These B29 Lys-modified human insulin conjugates (AcHI, PhacHI, AHI, and FHI) were characterized by mass analysis for corresponding molecular weight peaks (Figs. S7–S13), and purities were further checked by analytical HPLC (Figs. S2–S6). The yields of these newly synthesized insulin analogs were satisfactory. Further, we could collect unreacted insulin (approximately 10–14%) left out during this synthetic protocol using preparative HPLC. Soluble non-aggregated insulin is characterized by two negative minima in Circular Dichroism (CD) spectra around 208 nm and 222 nm, reflecting an overall *α*-helix-rich native insulin structure. Interestingly, even after modifications, the structural integrity of these modified insulin analogs viz. AcHI, PhacHI, AHI, and FHI were intact and are comparable to α-helix-rich native human insulin (HI) (Fig. S22). It is worth noting that while slight variations in ellipticity magnitude were observed in the CD spectra of all newly synthesized insulin analogs compared to native insulin, the consistent presence of negative bands at 208 and 222 nm confirmed that chemically modified analogs retained a predominant α-helical secondary structure, akin to native insulin (Fig. S22).

### Comparative thermostability of chemically modified insulin analogs

**Fig. 2:**
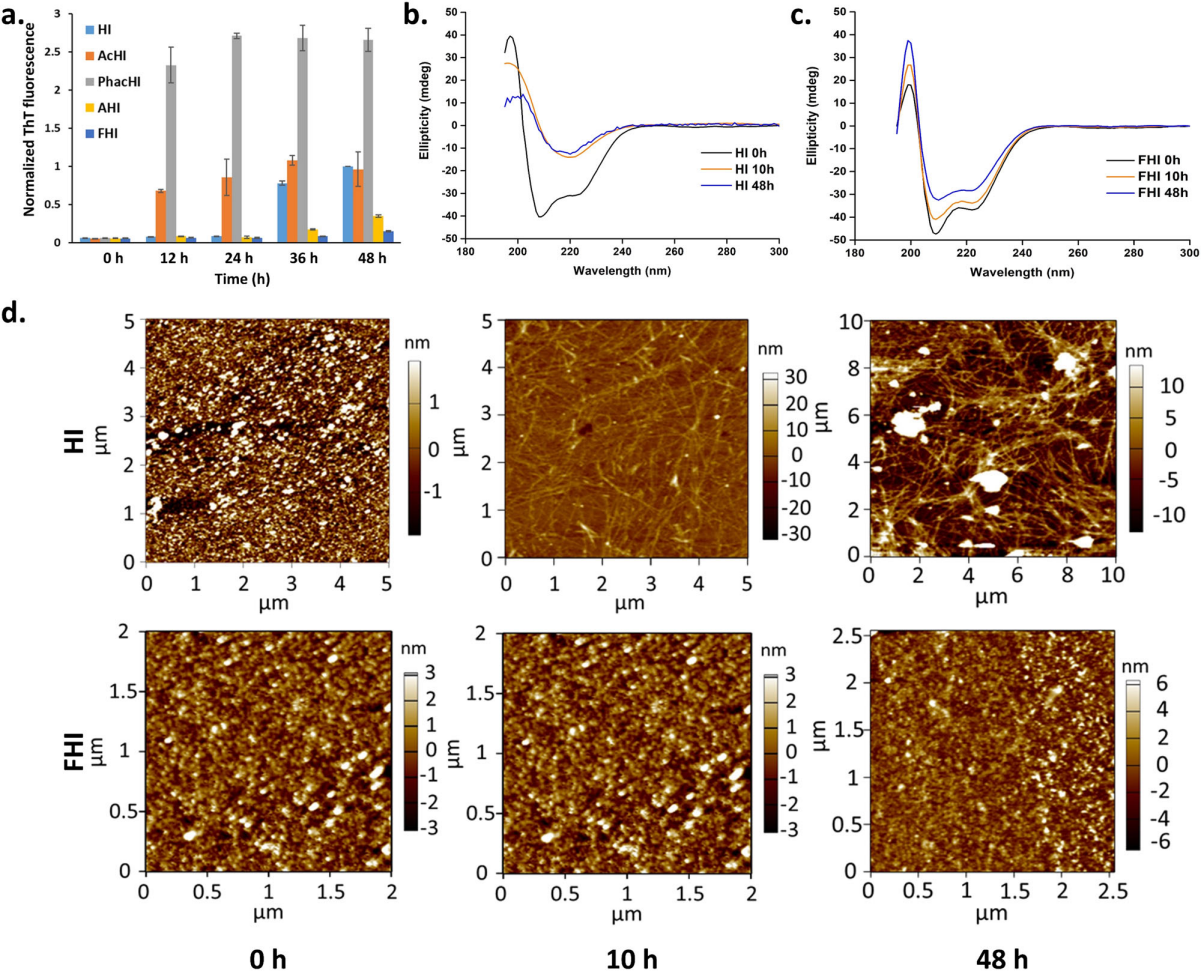
Comparative thermostability of chemically modified insulin analogs. **a** Normalized ThT fluorescence intensity (λ_em_) at 488 nm of HI and its derivatives (AcHI, PhacHI, AHI, and FHI). Data are represented as the mean of each group (*N* = 3) for each time point. Error bars represent the standard deviation; Circular Dichroism (CD) spectroscopy of (**b**) HI and (**c**) FHI, all CD experiments were carried out at 25 ± 0.1 °C using quartz cuvette with a path length of 1 mm; **d** Atomic Force Microscopy (AFM) micrographs of HI and FHI mounted over glass slide, incubated in 0.1 N HCl water (pH 1.6) containing 25 mM NaCl at 65 °C in different time intervals at concentration of 0.5 mg/mL (85 µM).

Comparative thermostability of HI, AcHI, PhacHI, AHI, and FHI was then evaluated by Thioflavin T (ThT) fluorescence assay (Figs. 2a, S23). ThT dye is a benzothiazole dye capable of binding specifically to protein aggregates, leading to an exponential increase in its fluorescence. To assess the propensities for fibrillation, insulin and its analogs (HI, AcHI, PhacHI, AHI, and FHI) were individually incubated in 1X PBS buffer (pH 7.4) amyloidogenic condition (at 65 °C) for 48 h and their fibrillation profiles were studied by measuring the intensity of ThT fluorescence (Fig. S23). The insulin analog FHI, in which phenylalanine is conjugated to the side chain amino group of B29 Lys, was the most effective in delaying the fibrillation process (Fig. S23). Further, incubation of insulin and its analogs in a more amyloidogenic condition, viz. 0.1 N HCl water (pH 1.6) containing 25 mM NaCl at 65 °C also revealed the highest resistance of FHI to fibrillation (Fig. 2a).

Masking the ε-amino group of B29 Lys with acetyl or phenylacetyl group increases the fibrillation propensity of insulin, indicating the importance of this ε-amino group in providing stability to insulin. Regenerating a new amino group at a distant place by conjugating ε-amine of B29 Lys with amino acids alanine and phenylalanine significantly reduces insulin fibrillation propensity (Figs. 2a, S23). Thus, a distant shift of the side chain amine by conjugating ε-amine of B29 Lys with an aromatic amino acid enhances the thermostability of insulin.

CD spectroscopy of insoluble insulin fibrils of cross-β sheet rich structure exhibits a negative peak around 218 nm. Heat-mediated transformation of *α*-helix-rich soluble native human insulin towards β-sheet rich fibril was seen around 10 h of incubation at 65 °C (in pH 1.6, 25 mM NaCl) (Fig. 2b). Whereas FHI maintained its native helical conformation even after 48 h of incubation (Fig. 2c). It is known that zinc facilitates insulin hexamer formation, thereby enhancing its structural stability^51^. Human insulin exhibits a distinct negative peak at 276 nm in its CD spectra, a characteristic feature of its dimeric and hexameric states, whereas, insulin monomers lack this peak^52^. We conducted near-UV CD spectroscopy by adding zinc to FHI and by comparing it with HI. Both HI and FHI displayed a negative peak at 276 nm, which intensified upon zinc addition (Fig. S30). Gradually increasing the temperature led to hexamer dissociation into monomeric populations, with a complete disappearance of 276 nm peak (at 80 °C), indicating that both HI and FHI were present in the monomeric state at this temperature (Fig. S30). We recorded ellipticities for HI and FHI by gradually increasing the temperatures from 5 to 110 °C, at 222 nm. A plot of ellipticity values versus temperature was used to calculate their melting temperatures (T_m_), which were found to be 68.72 and 76.74 °C, for HI and FHI, respectively (Fig. S31). Comparative fibrillation propensity of HI and FHI were then evaluated with atomic force microscopy (AFM) of incubated insulin samples (Fig. 2d). In amyloidogenic conditions, HI form insoluble fibrils of micro-meter ranges after 10 h of incubation, whereas FHI retained non-fibrillar state after prolonged incubation for 48 h (Fig. 2d). The results from HPLC and mass spectral analyses showed lack of degradation of FHI after 48 h of heating at both pH 1.6 (Figs. S32, S34, S35) and 7.4 (Figs. S33, S36, S37). In contrast, under the same conditions, complete degradation of HI occurred indicating enhanced thermostability of FHI.

### Comparative in vitro bioactivity of the insulin analog FHI

**Fig. 3:**
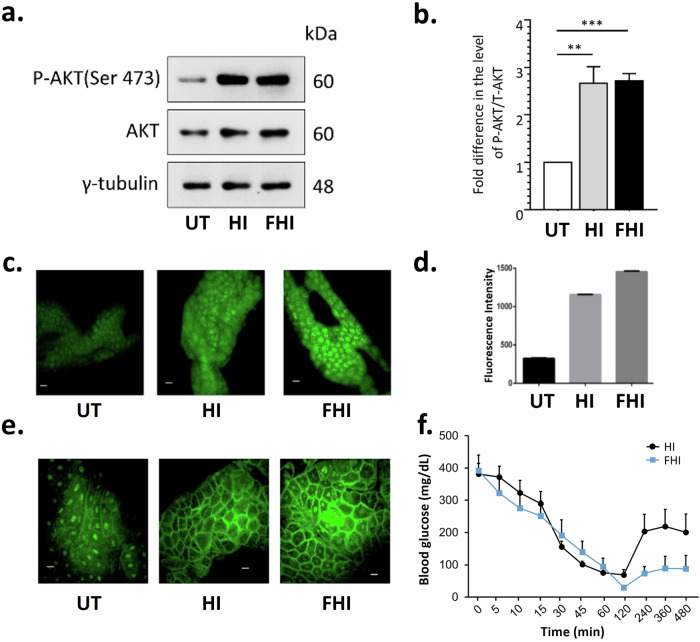
Comparative in vitro, ex vivo, and in vivo bioactivity. **a** Representative immunoblots showing the relative levels of Phosphorylated-AKT (Ser 473) in the HEK293T cell lines that are untreated (UT) or treated with 0.5 μM of human insulin HI or FHI for 30 min. γ-tubulin served as the loading control. **b** Bar diagram showing the fold change in intensities (measured by densitometry analysis) of Phosphorylated-AKT (P-AKT) normalized to total AKT (T-AKT) levels in HI or FHI treated HEK293T cells as compared to the untreated (UT) control (*N* = 4; *t-*test; **p ≤ 0.01 ***p ≤ 0.001, Error bars represent SE of mean); **c** Confocal microscopic images of 2-NBDG uptake in the explanted fat body of mid-third instar *Drosophila* larvae incubated with HI and FHI insulins as compared to their untreated control (UT) (Scale bar = 20 μm) and **d** quantification of 2-NBDG fluorescence. **e** Confocal microscopic images of mid-third instar larval fat body from transgenic *Drosophila* carrying GFP-tagged GRP1, also called tGPH. It displays nuclear localization when the fat body cells are not exposed to insulin, as in starved larvae. Upon incubation with HI and FHI insulins, tGPH fluorescence was characteristically seen in the membrane. Scale bar = 20 μm; **f** Blood glucose level in STZ treated C57BL/6 J wild-type mice after subcutaneous injection (1 µg/100 uL per 30 gm of mice) of Human insulin (HI) and the modified insulin FHI. Data is represented as the mean of each group (*N* = 5) for each time point. Error bars represent the SE of the mean.

Further, we validated the biological activity of the insulin analog FHI in the Human Embryonic Kidney cell line, HEK293T^53,54^. Insulin-mediated activation of the insulin receptors triggers a signaling cascade responsible for the phosphorylation of its downstream target Protein Kinase B (PKB also known as AKT) at the Ser473 and Thr308 residues^55^. Thus, to estimate the activity of the newly synthesized insulin analog FHI, we treated the HEK293T cells and checked the phosphorylation status of AKT at the Ser473 residue. We observed that upon treatment with FHI, HEK293T cells displayed increased levels of AKT-Ser473 phosphorylation similar to the wild-type insulin variant (HI) when compared to the untreated controls (UT) (Figs. 3a, b, S38), suggesting that chemical modifications in FHI do not alter the insulin signaling cascade.

### HI and FHI induce glucose uptake in *Drosophila* cells

Comparative ex vivo efficacy of the human insulin and its modified variant FHI were then screened using starved *Drosophila* larval tissue. A high conservation of genes, molecules, and pathways makes *Drosophila* an ideal model for studying insulin metabolism ex vivo^56,57^. The insulin receptor (InR), the insulin receptor substrate (CHICO), and the phosphoinositide 3-kinase (PI3K) signaling pathway are conserved from humans to *Drosophila* flies^58^. *Drosophila* insulin-producing cells (IPCs) secrete DILPs (*Drosophila* insulin-like peptides) in nutrient surplus conditions—as do mammalian insulin—which bind to insulin receptors present on the fat body (reminiscent of mammalian adipose tissue and liver) and muscles to regulate glucose uptake, utilization, and its storage. Due to its conservation, *Drosophila* responds to exogenous human insulin, triggering its endogenous signaling cascade^59–61^. When starved for 12 h, the endogenous insulin levels go down in the *Drosophila* larvae; a consequent lowering of glucose uptake is revealed from the near absence of uptake of a fluorescent analog of glucose, 2-NBDG (Figs. 3c, d). However, adding exogenous HI and FHI restores 2-NBDG uptake (Fig. 3c, d).

### HI and FHI induce the PI3K signaling pathway in *Drosophila*

Signaling cascade downstream of insulin is also conserved between mammals and flies^57^. In *Drosophila*, feeding increases circulatory glucose levels and stimulates the insulin-producing cells (IPCs) to release DILPs that bind to insulin receptors and activate downstream signaling like the PI3K pathway. Upon activation, PI3K phosphorylates inositol lipids and generates several secondary messengers, among which the most critical one is phosphatidylinositol-3,4,5-P_3_ (PIP_3_), which mainly resides in the plasma membrane. The pleckstrin homology (PH) domain of the General Receptor for Phosphoinositides-1 (GRP1) binds specifically to PIP3, leading to the recruitment of GRP1 at the cell membrane^62,63^. Transgenic *Drosophila* carrying GRP1 fused GFP, also termed tGPH, provides a ready and reliable assay for activation of PI3K signaling. For instance, cell membrane-bound tGPH fluorescence declines in fat body explanted from starved larvae and incubated in PBS (Fig. 3e). By contrast, fat body explanted from starved larvae and then exposed to HI and FHI ex vivo display robust membrane-bound tGPH fluorescence, thereby revealing activation of the endogenous PI3K signaling (Fig. 3e). The comparable efficacy of FHI and HI in activating PI3K signaling downstream of insulin shows that B29 lys modification does not alter its signaling potency.

### In vivo, glucose lowering activity of FHI in the diabetic mice model

The efficacy of the newly synthesized insulin derivative FHI was also screened in vivo in a Streptozotocin (STZ) induced Type I diabetes mouse model. STZ is an antibiotic that ablates the insulin-producing beta cells of the pancreas, thus removing the endogenous source of insulin, causing an increased influx of glucose in the bloodstream^64^. We observed that treatment with the modified insulin FHI significantly reduced the blood glucose levels in the STZ-induced diabetic mice without any observable phenotypic alterations (motor impairment, mortality) and was comparable to that of the wild-type human insulin treatment (Fig. 3f). The result further confirmed that the newly modified insulin analog FHI was equally active as HI in vivo.

### In vitro and in vivo bioactivity of FHI after prolonged exposure to higher temperature

**Fig. 4:**
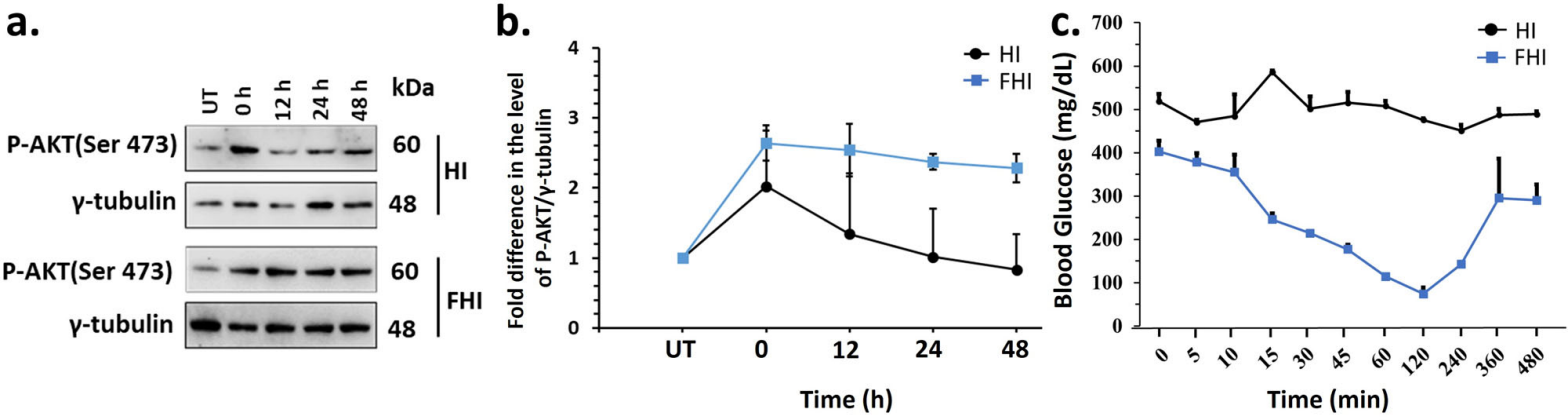
In vitro and in vivo bioactivity of FHI after prolonged exposure to higher temperature. **a** Representative immunoblots showing the relative levels of P-AKT (Ser473) in the HEK293T cell lines that are untreated (UT) or treated for 30 min with 0.5 µM of HI or FHI incubated at 65 °C for different time points as indicated. γ-tubulin served as the loading control; **b** Plot showing the change in the activity of P-AKT in the incubated HI or FHI-treated HEK293T cells as compared to the untreated (UT) control at the indicated time points. (*N* = 3, Error bars represent SE of mean); **c** Blood glucose level in STZ-treated C57BL/6 J wild-type mice after subcutaneous injection (1 µg/100 µL per 30 gm of mice) of heated human insulin and the modified insulin FHI. Heating was carried out in 0.1 N HCl water (pH 1.6) containing 25 mM NaCl at 65 °C for 12 h and 48 h for HI and FHI respectively. Data are represented as the mean of each group (*N* = 3) for each time point. Error bars represent the SE of the mean.

Commercially available insulin is known to lose its activity upon heat exposure and during storage in refrigeration for a longer duration^65^. To test whether the modified insulin analog FHI could maintain its biological activity upon protracted heat treatment, we incubated both HI and FHI at 65 °C for 12, 24, and 48 h. We checked the status of AKT Ser473 phosphorylation upon their treatments in the HEK293T cells (Figs. 4a, b, S39). Figures 4a and 4b show that the wild-type human insulin (HI) lost most of its bioactivity upon 12 h of incubation at 65 °C. By contrast, FHI retained its functional activity and significantly increased the phosphorylation levels of AKT Ser473 compared to untreated control (UT) even after 48 h of heat treatment. Similarly, we evaluated insulin bio-efficacy upon prolonged refrigeration at 4 °C and observed that FHI maintains its bioactivity even after two months of refrigeration. In contrast, human insulin completely lost activity during this storage condition (Fig. S24). These results suggest that FHI is far more stable than HI at high temperatures and cold storage conditions. Next, the in vivo bioefficacy of the heated HI and FHI samples was screened in the STZ-treated diabetic mice model to determine their thermostability. FHI sample on heating at 65 °C for 48 h retained its efficacy to lower the blood glucose level, which was lost in the HI following 12 h heating (Fig. 4c).

### Molecular underpinning of enhanced stability of FHI

**Fig. 5:**
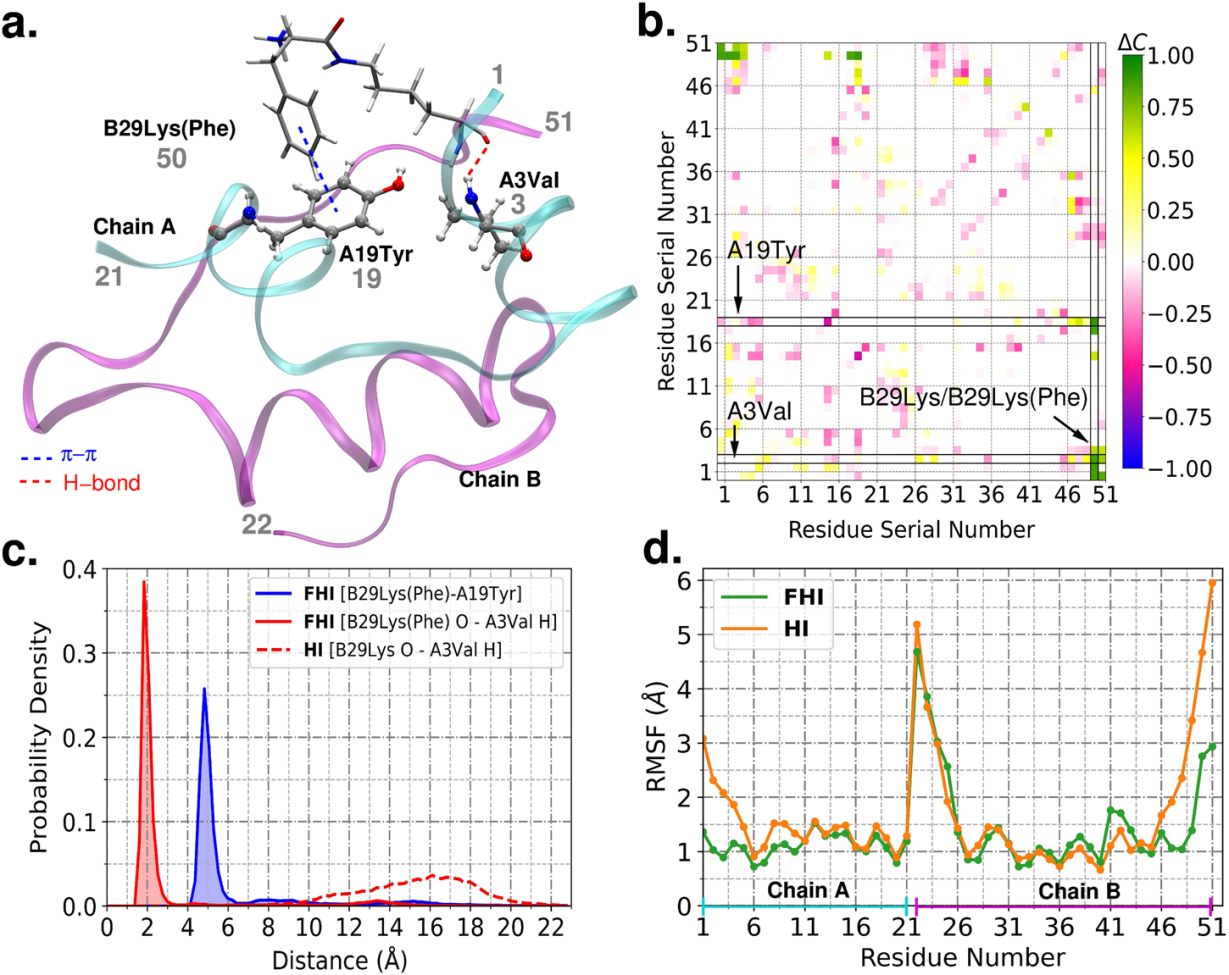
Molecular dynamics (MD) simulations study of FHI and HI. **a** Structure of FHI, where B29 Lys(Phe)…A19Tyr, B29 Lys(Phe)…A3Val interactions are shown. Atom color code: H (white), C (gray), O (red), and N (blue). Residues A1 to A21 are assigned residue serial numbers from 1 to 21, and residues B1 to B30 are given residue serial numbers from 22 to 51. Some of the residue serial numbers are indicated (gray); **b** Difference in the population of contact maps of residues in FHI and HI (ΔC = C_**FHI**_ – C_**HI**_). Population, C, of a contact in the contact map is graded from 1.0 (high) to 0.0 (low). High positive (negative) values of ΔC correspond to an increase (decrease) in the population of any contact upon conjugation of phenylalanine in B29 Lys residue. **c** The probability distributions of the distance between the backbone carbonyl oxygen of B29 Lys/B29 Lys(Phe) and backbone amide hydrogen of A3Val, and the distance between the center of mass of aromatic rings of B29 Lys(Phe) and A19Tyr. **d** Root mean square fluctuations (RMSFs) of residues of FHI as compared to that of HI.

Molecular dynamics (MD) simulations for monomeric structures of both HI and FHI were executed to decipher the molecular interactions responsible for the additional stability of FHI (Fig. 5). The contact map analysis of HI and FHI showing contact between all non-hydrogen atoms of the residue within the cut-off distance of 4.5 Å was initially performed (Figs. 5b, S25). In the contact map of FHI, phenylalanine conjugated B29 Lys [B29 Lys(Phe)] residue shows significant proximity with the residues A1Gly, A2Ile, A3Val, A4Glu, A18Asn, and A19Tyr for most of the 1 µs long MD simulation (Figs. 5b, S25). B29 Lys formed no stable contacts with any other residues in the native insulin (Fig. S25). T-shaped π-π (CH/π hydrogen bonding) interaction formed between the aromatic ring of the phenylalanine conjugated lysine [B29 Lys(Phe)] and A19 tyrosine residue was observed during the entire MD trajectory of FHI (Fig. 5a, c). Also, stable hydrogen bonding was seen between the backbone carbonyl oxygen of B29 Lys(Phe) and the amide backbone hydrogen of A3Val residue (Fig. 5a, c). The probability distributions of these distances have only one prominent peak and were around 5 Å and 2 Å, respectively (Fig. 5c). These interactions were absent in the MD simulations trajectory of native insulin (Figs. 5c, S27). Rest we then analyzed the root mean square fluctuations (RMSFs) of HI and FHI terminal residues. The RMSFs of the terminal residues A1Gly, A2Ile, A3Val, B26Tyr, B28Pro, B29 Lys(Phe), and B30Thr of FHI are much less than that of corresponding HI residues. This outcome implies that the fluctuation in FHI is restricted compared to the terminal residues of HI (Fig. 5d). The total number of H-bonds and π-π interactions were computed (Fig. S28). A noticeable increase in the number of π-π aromatic interactions can be seen in the case of FHI as a phenyl ring is added in the insulin monomer in the form of B29Lys(Phe) residue. To further quantify and establish the differences, we computed the total electrostatic and van der Waals energy for the HI and FHI proteins (Fig. S29). Both quantities are lower in the case of FHI, reconfirming the enhanced stability of FHI. Thus, the combined effect of π-π and H-bonding interactions introduced by B29 Lys(Phe) makes the structure of FHI relatively more stable than HI by minimizing the terminal chain distortions.

## Discussion

We aimed to synthesize and evaluate the thermostability of four insulin analogs created through selective conjugation at the ε-amine of B29 Lysine in insulin. This position has been previously explored for the conjugation of insulin to albumin-binding side chains derived from octadecanedioic or icosanedioic acid and with fatty acids through glutamic acid spacers to improve pharmacokinetic profiles^66–68^. Prior studies on B29 modifications were mainly focused on pharmacological efficacies like duration of action, cell selectivity, glucose sensitivity, etc. (Table S1)^46^. For instance, insulin degludec, a long-acting insulin, displays oligomerization induced by B29 Lys-conjugated hexadecanedioic acid. Another analog, insulin detemir, achieves a prolonged metabolic effect by binding to albumin via its B29 Lys-conjugated myristic acid chain. The hepatoselective action of an insulin analog, insulin-327, stems from a B29 Lys modification with a fatty diacid. Glucose-responsive analogs were also created using selective phenylboronic acid conjugation at B29 Lys^46^. However, the thermal stability of insulin, a crucial property, has been overlooked in these studies. Although recent efforts have attempted to acetylate B29Lys position along with the amine groups of the N-terminal amino acids of both the A and B chains to achieve better thermostability, these attempts afforded triacetylated version of human insulin rather than a single site-specific acetylation at B29Lys position^45^. The modification procedure, characterization, and experimental conditions described in the current study are far more rigorous and superior in terms of chemical modification, biological evaluation, and computations to the previously reported method, including a comprehensive MD simulation insight^45^. (Table S2).

A minimal modification procedure described here presented human insulin analogs with enhanced stability and, crucially, resistance to thermal degradation. Phenylalanine-conjugation at ε-amine of B29 lysine in insulin displayed here stabilizes the structural framework of FHI by minimizing fluctuations in amino acid residues, particularly in the A-chain *N*-terminal and B-chain C-terminal. This stabilization is facilitated through additional aromatic π-π interactions and hydrogen bonding. The insights gained from these simulations also present a promising avenue for newer insulin analogs with improved thermostability. Importantly, this modification was found to be well-tolerated in biological systems. FHI exhibited nearly identical if not improved, activity compared to human insulin in in vitro, ex vivo, and in vivo experiments.

In summary, we present a remarkable example of insulin chemical modification that offers a prototype for the next generation of highly thermostable and pharmacologically active insulin analogs. Given the preclinical efficacies, these B29 Lys-modified insulin analogs offer important candidates for eventual clinical trials. The ease of synthesis and scale-up makes the present breakthrough worthy of closer scrutiny towards overcoming cold-chain and storage-linked challenges of insulin injectables.

## Methods

### General information

Human insulin (Cat. No. 91077c) was purchased from Sigma Aldrich. Trifluoroacetic acid (TFA), Boc-anhydride, and N-Hydroxy succinimide (NHS) were purchased from Avra Synthesis Pvt. Ltd. (Hyderabad, India). Dry *N*, *N-*dimethylformamide (DMF), and HPLC grade acetonitrile (ACN) were purchased from Finar Chemicals and S. D. Fine-Chem Pvt. Ltd. respectively. *N*-Ethyl-*N’*-(3-dimethylaminopropyl)carbodiimide hydrochloride (EDC.HCl) and Thioflavin T (ThT) were purchased from Sisco Research Laboratories Pvt. Ltd. (SRL). Reactions were monitored by thin layer chromatography (TLC) carried out on readymade TLC silica gel 60F_254_ plates (Merck, Dermstadt, Germany) and compounds were visualized with UV light at 254 nm. ^1^H NMR spectra were recorded on JEOL-JNM spectrometer operating at 400 MHz at 25 °C using 2–10 mM concentration in appropriate solvents using TMS as an internal standard or the solvent signals as secondary standards and the chemical shifts (δ) are shown in ppm scales. Multiplicities of NMR signals are designated as s (singlet), d (doublet), and m (multiplet, for unresolved lines). ^13^C NMR spectra were recorded at 100 MHz with complete proton decoupling. Mass spectra were recorded on ESI-MS or MALDI-ToF-MS. ThT fluorescence was recorded on SpectraMax iD3 Multi-Mode Microplate Reader. Circular Dichroism (CD) spectra were recorded on a JASCO J-815 CD spectrometer. Microscopy images were taken by Leica SP6 confocal microscope, processed using LAS AF software, and analyzed with ImageJ software.

### Synthesis of B29 Lys Boc-phenylalanine conjugate human insulin (Boc-FHI)

Human insulin (20 mg, 3.44 µmol) solubilized in 10 mM HCl solution (2 mL) and to this 14 mL of 100 mM carbonate-bicarbonate buffer (pH 10) was added and adjusted to the final pH of the reaction mixture to pH 10. Boc-Phe-OSu (3.74 mg, 3 equiv., 10.34 µmol) was dissolved in 100 µL of dimethylformamide and added to the reaction mixture. The addition of extra Boc-Phe-OSu was carried out at 2 h intervals twice. The reaction was carried out at room temperature with moderate stirring. The desired product formed would be Boc-Phenylalanine-human insulin (Boc-FHI). The extent of the reaction was screened through analytical high-performance liquid chromatography using a C18 reverse-phase analytical column.

The formation of the product can be analyzed through the appearance of a new peak around 13.5 min in addition to the peak of human insulin that appeared around 11.5 min in C18 analytical HPLC column elution. Reaction mixture was lyophilized and the dried reaction mixture was dissolved in 4 mL of HPLC grade water. This solution was injected in the C18 reverse phase preparative column and the corresponding product (Boc-FHI) peak was collected. Acetonitrile present in the collected fraction was evaporated at 40 °C under reduced pressure and afterward, the sample was lyophilized and we got Boc-FHI with an overall yield of 85.5% (17.8 mg, 2.94 µmol) with separately recovered 14% non-reacted human insulin. Boc-FHI was characterized through MALDI-MS analysis by observing the peak at 6058.916 Da (Fig. S8).

### Synthesis of B29 Lys Boc-alanine conjugated human insulin (Boc-AHI)

Synthesized by the same method described for Boc-FHI. Yield (16.91 mg, 2.83 µmol, 82.3%). Boc-AHI was characterized through MALDI-MS analysis by observing the peak at 5970.740 Da (Fig. S9).

### Synthesis of B29 Lys acetyl conjugated human insulin (AcHI)

Synthesized by the same method described for Boc-FHI. Yield (15.91 mg, 2.72 µmol, 79.06%). AcHI was characterized through ESI-MS analysis by observing the peak at 5843.587 Da (Fig. S10).

### Synthesis of B29 Lys phenylacetyl conjugated human insulin (PhacHI)

Synthesized by the same method described for Boc-FHI. Yield (16.76 mg, 2.83 µmol, 82.26%). PhacHI was characterized through ESI-MS analysis by observing the peak at 5925.19 Da (Fig. S11).

### Synthesis of B29 Lys phenylalanine conjugated human insulin (FHI)

Boc deprotection was carried out by treating Boc-FHI (17.8 mg, 2.94 µmol) with 2 mL of trifluoroacetic acid at room temperature for 20 min. Trifluoroacetic acid was evaporated at 40 °C under reduced pressure and afterward, Boc deprotected insulin derivative was precipitated with anhydrous diethyl ether. Solvent was drained off to recover precipitated insulin. Afterward, 2 mL of HPLC-grade water was added to solubilize insulin and lyophilized to get phenylalanine conjugated human insulin derivative (FHI) as a white powder with almost quantitative yield. Formation of FHI was analyzed through the appearance of an elution peak at a retention time of 11.3 min in the analytical C18 HPLC column. FHI was characterized through ESI-MS analysis by observing the peak at 5954.30 Da (Fig. S12).

### Synthesis of B29 Lys alanine conjugated human insulin (AHI)

Synthesized by the same method described for FHI. Yield (quantitative). AHI was characterized through ESI-MS analysis by observing the peak at 5878.17 Da (Fig. S13).

### Circular dichroism spectroscopy

Conservation of the structural integrity even after protein modification was confirmed by circular dichroism (CD) spectroscopy. Spectra were collected using JASCO J-815 CD spectrometer and a quartz cuvette of 1 mm path length. CD spectra of proteins (0.5 mg/mL or 85 μM) were collected between 195 to 300 nm and each spectrum represents the average of three scans. To avoid any instrumental baseline drift contributed by the working buffer, the background values were subtracted from each individual sample measurement.

### Thioflavin T fluorescence assay

Thioflavin T (ThT) is an aggregation-indicating dye that shows an increase in its fluorescence upon binding with protein aggregates. 0.5 mg/mL (85 μM) solution of human insulin (HI) and its derivatives (AcHI, PhacHI, AHI, and FHI) have been prepared in PBS buffer (pH 7.4). These solutions were incubated at elevated temperature (65 °C). A total of 30 μL of ongoing incubated protein sample was taken out at different time points and mixed with 200 μL of 50 μM Thioflavin T (ThT) dye solution prepared in PBS buffer (pH 7.4) with 10 min of incubation in the dark. Emission spectra were collected from 450 to 560 nm with excitation at 410 nm (λ_ex_) using a Corning® 96 well clear flat bottom black polystyrene microplate in SpectraMax iD3 Multi-Mode Microplate Reader. The spectra were recorded at different time points i.e. 0 h, 12 h, 24 h, 36 h, and 48 h of incubation, and the graph was plotted with an emission value of 488 nm. Similarly, ThT assays were conducted for these samples at lower pH (0.1 N HCl water, pH 1.6) with an additional salt concentration of 25 mM of NaCl.

### Time-dependent circular dichroism spectroscopy

CD spectra were collected using JASCO J-815 CD spectrometer and a quartz cuvette of 1 mm path length. Spectra of 0 h, 10 h, 48 h incubated samples of HI (85 μM) and FHI (85 μM) were collected between 195 to 300 nm. Each spectrum represents the average of three scans. To avoid any instrumental baseline drift contributed by the working buffer, the background values were subtracted from each individual sample measurement.

### Atomic force microscopy & scanning electron microscopy

Comparative time-dependent visualization of fibrillation as well as its hindrance was carried out by atomic force microscopy (AFM) as well as scanning electron microscopy (SEM). 0.5 mg/mL (85 μM) of human insulin (HI) and phenylalanine-human insulin (FHI) samples were incubated separately in 0.1 N HCl water (pH 1.6) containing 25 mM NaCl at 65 °C. Afterward, incubated samples were diluted up to ~17 μM of final concentration before sample preparation. 10 µL were drop casted on glass surface at room temperature. All samples were allowed to air dry at room temperature for overnight followed by subsequent drying under vacuum for 30 minutes prior to scanning.

The samples were scanned with an atomic force microscope (Asylum Research, Oxford Instruments, MFP-3D Origin) at room temperature. Scanning was carried out under tapping mode with a force constant of 21 N/m. Silicon nitride cantilever from Nanosensors with following features was used; Resonance frequency: 170 kHz, Thickness: 7.0 µm, length: 225 µm, width: 38 µm. Field emission scanning electron microscopy (FESEM) images were acquired on ZEISS SUPRA 40 VP microscope, operating at 10 kV. The samples were gold coated for 1 min and then imaged with FESEM.

### Cell culture and immunoblotting

HEK293T cell lines were obtained from the National Center of Cell Sciences (NCCS, Pune, India) and were cultured in ‘Dulbecco’s modified ‘Eagle’s medium (Sigma–Aldrich), supplemented with 10% (v/v) fetal bovine serum (GIBCO International) and antibiotic-antimycotic solution (Sigma–Aldrich). The cells were grown under aseptic conditions in incubators with 5% CO_2_, 37 °C, and a humid environment. Cells with 80–90% confluency were passaged for maintaining the running culture.

To perform immunoblotting, the cells were harvested and lysed in SDS-DTT lysis buffer (60 mM Tris-pH 6.8, 2% SDS, 100 mM DTT), resolved on SDS-PAGE and the protein was transferred to the nitrocellulose membrane. The membranes were then blocked with 5% non-fat milk blocking buffer and were incubated with the specific primary and respective secondary antibodies using the manufacturer’s respective protocol. The protein bands were developed using a chemiluminescence detection kit (SuperSignal West PICO Chemiluminescent Substrate, Thermo Fisher Scientific India Pvt. Ltd., Mumbai, India) and imaged and quantified using a ChemiDoc imaging system (Biorad). The following antibodies were used in the current study: anti-γ-tubulin (T6557; IB; 1:10,000) from Sigma Aldrich, anti-P-AKT (#4051) and anti-AKT (#9272) from Cell Signaling Technology.

### Glucose uptake assay

Comparative ex vivo efficacy of the newly derived human insulin derivative (FHI) has been screened using starved drosophila larvae tissue. Fat body of mid-third instar larvae were dissected on ice and incubated with 0.5 μmol of human insulin (HI) or its derivative FHI in PBS in the presence of 0.25 mM 2-(N-(7-nitrobenz-2-oxa-1,3-diazol-4-yl)amino)-2-deoxyglucose (2-NBDG) (N13195, Invitrogen) for 15 min at room temperature. The tissues were washed with 1X PBS thrice, mounted in 80% glycerol; images were taken immediately in a Leica SP6 confocal microscope and processed using LAS AF software, and analyzed with ImageJ software. The fluorescence was quantified using ImageJ software.

### Downstream insulin signaling cascade

Transgenic tGPH drosophila flies were used to check insulin activity in which GRP1 is fused with a green fluorescent protein. Fat body of mid-third instar larvae was dissected on ice and incubated with 0.5 μmol of human insulin (91077 C, Merck) in PBS (or 1x PBS as a control) for 15 minutes at room temperature. The tissues were fixed in 4% paraformaldehyde in phosphate-buffered saline with 1% Triton X-100 (PBT) and mounted in 80% glycerol after washing thrice with PBT. Images were taken in Leica SP6 confocal microscope, processed using LAS AF software, and analyzed using ImageJ.

### Animal experiments

C57BL/6 J mice were used for animal experiments. The animal experiments were approved by the Institutional Animal Ethics Committee (IAEC) of IIT Kanpur (Protocol no: IITK/IAEC/2016/1048) and the experiments were performed according to the guidelines proposed by the Committee for the Purpose of Control and Supervision of Experiments on Animals, Govt. of India. All the experimental animals were maintained in the ‘institutes’ facility on a 12/12 light/dark cycle in a regulated temperature (25 °C) with ad libitum access to food and water.

### Blood glucose monitoring in diabetic mice model

In vivo activity of FHI was measured in a STZ-induced model for diabetes. Briefly, 2–3 months old male wild-type C57BL/6 J mice were treated with Streptozotocin (70 mg/kg for six days) and the vehicle. Blood glucose was monitored for a period of 3–4 days after the treatment regime using the glucometer (LifeScan Inc) to confirm the induction of diabetes in the mice. For the insulin tolerance test, mice were starved for 8 h and were injected with buffer (0.9% saline), HI, FHI subcutaneously at a dosage of 1 µg/100 µL per 30 gm of body weight. Blood glucose level was measured for 5, 10, 15, 30, 45, 60,120, 240, 360, 480 min post the injection using the glucometer (LifeScan Inc) and plotted as absolute values in mg/dL.

### Insulin activity on long cold storage or at elevated temperature

To test the efficacy of the modified insulin analog FHI as compared to that of HI, we either stored both HI and FHI at 4 °C for two months or heated for a period of 12, 24, 48 h. Serum-starved HEK293T cells were then treated with both HI and FHI at a concentration of 0.5 µmol for 30 min and the level of phosphorylated AKT was measured using western blot analysis for specific antibodies as a readout of insulin activity.

### Molecular dynamics simulations

Initial coordinates for the MD simulations were taken from the NMR structure of the human insulin monomer (PDB: 2JV1)^69^. The protonation state of residues was determined by the H++ server^70^, corresponding to a pH of 1.6. MD simulations were performed with AMBER ff14SB force-field^71^, and TIP3P water^72^ model at 338 K. GAFF force-field^73^ was used for B29 Lys(Phe) residue. Analysis was carried out using 1 μs MD trajectory of HI and FHI in NVT ensemble. More details are given in ESI Section 8.

### Reporting summary

Further information on research design is available in the Nature Portfolio Reporting Summary linked to this article.

### Supplementary information

Supplementary information

Description of Additional Supplementary Files

Supplementary data 1

Supplementary data 2

Supplementary data 3

Supplementary data 4

Supplementary data 5

Reporting Summary

## Data Availability

Data sharing is not applicable to this article as no datasets were generated or analyzed during the current study. (1) Supplementary information (Containing supplementary schemes, tables, figures etc.). (2) Supplementary data 1 (Contains excel file having numerical source data for graphs). (3) Supplementary data 2 (PDB file having initial MD simulation structure of HI). (4) Supplementary data 3 (PDB file having final MD simulation structure of HI). (5) Supplementary data 4 (PDB file having initial MD simulation structure of FHI). (6) Supplementary data 5 (PDB file having final MD simulation structure of FHI).
