# Supplementary material for: Synthesis of a highly thermostable insulin by phenylalanine conjugation at B29 Lysine
Source: Commun Chem. 2024 Jul 23;7:161. doi: 10.1038/s42004-024-01241-z (PMC11266353; doi:10.1038/s42004-024-01241-z)
Supplement: Supplementary file 1 — Supplementary information [file 42004_2024_1241_MOESM1_ESM.pdf]

Synthesis of a highly thermostable insulin by phenylalanine conjugation at B29 Lysine

Shantanu Sen,[‡a] Rafat Ali,[‡a] Akanksha Onkar,[b,c] Shivani Verma,[a] Quazi Taushif Ahmad,[b] Pratibha Bhadauriya,[b] Pradip Sinha,[b] Nisanth N. Nair,[a] Subramaniam Ganesh,[b,d] and Sandeep Verma*[a,d,e]

‡ These authors contributed equally.

a. Department of Chemistry, Indian Institute of Technology Kanpur, Kanpur 208016, UP, India. E-mail: sverma@iitk.ac.in.

b. Department of Biological Sciences & Bioengineering, Indian Institute of Technology Kanpur, Kanpur 208016, UP, India.

c. Current address: Department of Laboratory Medicine, University of California San Francisco, San Francisco 94143, CA, USA.

d. Mehta Centre for Engineering in Medicine, Indian Institute of Technology, Kanpur 208016, UP, India.

e. Gangwal School of Medical Sciences and Technology, Indian Institute of Technology, Kanpur 208016, UP, India.

Contents

		Pages
1.	**Synthesis protocol**	S2
2.	**HPLC analysis**	S4
3.	**Mass spectrometry**	S6
4.	**Copies of ^{1}H and ^{13}C NMR spectra**	S8
5.	**Circular dichroism (CD) spectroscopy**	S12
6.	**ThT assay**	S13
7.	**Insulin activity on long cold storage**	S13
8.	**Molecular Dynamics Simulations**	S14
9.	**Oligomerization status of HI and FHI**	S17
10.	**Melting temperature of HI and FHI**	S17
11.	**HPLC analysis of the samples after heat treatment**	S18
12.	**Mass analyses of the samples after heat treatment**	S20
13.	**B29 Lys modifications in insulin**	S22
14.	**Comparison of current work with previous study**	S22
15.	**Uncropped immunoblots**	S24
16.	**Supplementary references**	S25

1. Synthesis protocol

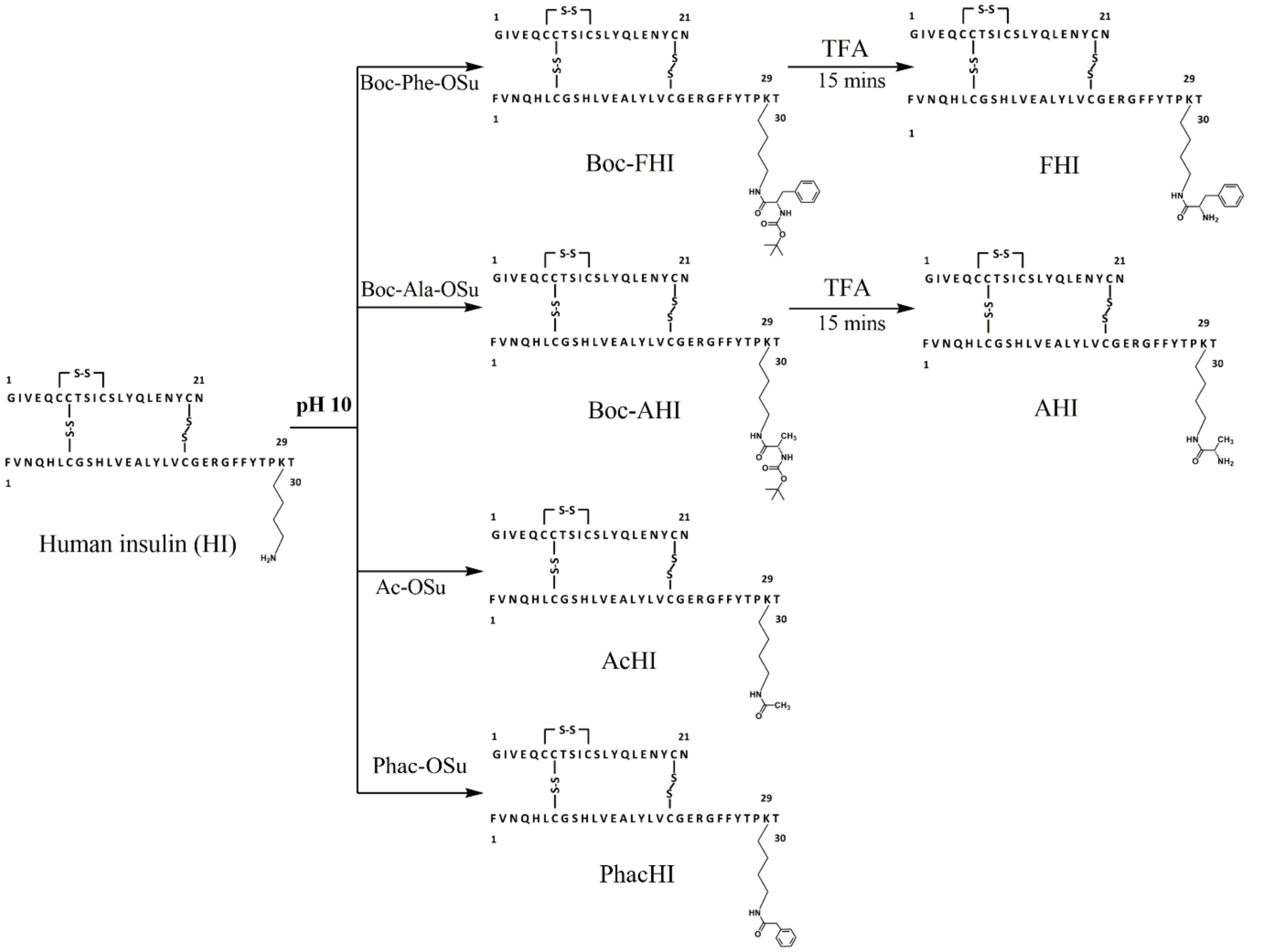

Scheme S1: Synthetic protocol for **FHI**, **AHI**, **AcHI** and **PhacHI**.

1.1. Synthesis of Boc-Phe-OSu:

L-Phenylalanine (2 gm,12 mmol) was suspended in distilled 1,4-Dioxane (30 mL) and distilled water (20 mL) and the resulting suspension was kept at 0 ºC. To this 1M NaOH solution in water (15 mL) was added and finally Di-*tert-butyl* dicarbonate (1.2 equiv, 3.05 gm, 14 mmol) was added under cold condition. The reaction mixture was allowed to come to room temperature and stirred for 3 h at room temperature. After completion of the reaction (monitored through TLC), 1,4-dioxane was evaporated under reduced pressure. The water layer was extracted with ethyl acetate to remove the excess

$(Boc)_2O$. The aqueous layer was then acidified with 1N HCl to pH 4-5. After acidification aqueous layer was extracted with ethyl acetate (2 times) and combined organic layer was washed with brine solution followed by drying of organic layer over anhydrous sodium sulphate. Finally, the solvent was evaporated under reduced pressure to get Boc-Phe-OH as white solid which is used directly in the next step without purification.

Boc-L-Phenylalanine (1.6 gm, 6.05 mmol) and *N*-hydroxysuccinimide (1.2 equiv., 0.83 gm, 7.26 mmol) were dissolved in dry dichloromethane and cooled to 0 ºC. To this small amount of tetrahydrofuran was added to achieve complete solubility. To this 1-(3-Dimethylaminopropyl)-3-ethylcarbodiimide hydrochloride or EDC.HCl (1.5 equiv., 1.73 gm, 9.075 mmol) was added directly to the reaction under cold condition. The reaction was stirred for 1 h at 0 °C and then stirred overnight at RT under anhydrous condition. Reaction was monitored using TLC and upon completion solvents including dichloromethane as well as tetrahydrofuran were evaporated. The residual was then dissolved in ethyl acetate and afterwards washed with water. Collected organic layer was treated with brine followed by drying of organic layer over anhydrous sodium sulphate and evaporated under reduced pressure to give Boc-Phe-OSu as white solid. yield (1.7 gm, 4.8 mmol, 79.3 %); ^{1}H NMR (400 MHz, $CDCl_3$) δ/ppm = 7.3-7.26 (m, 5H), 4.95-4.87 (m, 2H), 3.34-3.29 (m, 1H), 3.21-3.18 (m, 1H), 2.86 (s, 4H), 1.41 (s, 9H); ^{13}C NMR (100 MHz, $CDCl_3$) δ/ppm = 168.66, 167.93, 159.34, 134.63, 129.78, 128.76, 127.62, 80.65, 52.68, 38.22, 28.30, 25.67.

1.2. <u>Synthesis of Boc-Ala-OSu:</u>

Synthesized by the same method described for Boc-Phe-OSu. Yield (1.4 gm, 5.2 mmol, 85.9 %).^{1}H NMR (400 MHz, $CDCl_3$) δ/ppm = 5.00 (bs, 1H), 4.71-4.69 (m, 1H), 2.85 (s, 4H), 1.57 (d, *J* = 7.24 Hz, 3H), 1.46 (s, 9H); ^{13}C NMR (100 MHz, $CDCl_3$) δ/ppm =169.12, 168.73, 154.70, 80.59, 47.74, 28.32, 25.65, 18.77.

1.3. <u>Synthesis of Ac-OSu:</u>

Acetic acid (1.96 mmol) and *N*-hydroxysuccinimide (0.361 gm, 3.14 mmol) were dissolved in dry dichloromethane (3 mL) and cooled to 0 ºC. To this 1-(3-Dimethylaminopropyl)-3-ethylcarbodiimide hydrochloride or EDC.HCl (0.061 g, 3.14 mmol) was added directly to the reaction under cold condition. The reaction was stirred for 1 h at 0 °C and then stirred overnight at RT under anhydrous condition. Reaction was monitored using TLC and upon completion extra dichloromethane was added and afterwards washed with water. Collected organic layer was washed with saturated bicarbonate solution. Collected organic layer was further treated with brine solution followed by drying of organic layer over anhydrous sodium sulphate and evaporated under reduced pressure to give succinimidy ester of acetic acid (Ac-OSu) as white solid. Yield (0.246 gm, 1.57 mmol, 80.1 %).^{1}H NMR (400 MHz, $CDCl_3$) δ/ppm = 2.84 (s, 4H), 2.35 (s, 3H); ^{13}C NMR (100 MHz, $CDCl_3$) δ/ppm = 169.23, 165.68, 25.64, 17.66.

1.4. <u>Synthesis of Phac-OSu:</u>

Succinimidyl ester phenyl acetic acid (Phac-OSu) by the same method described for Ac-OSu. Yield (0.382 gm, 1.64 mmol, 83.67 %).^{1}H NMR (400 MHz, DMSO-d6) δ/ppm = 7.35-7.26 (m, 5H), 4.06 (s,

2H), 2.76 (s, 4H); ^{13}C NMR (100 MHz, DMSO-d_6) δ/ppm = 170.70, 167.95, 132.88, 129.89, 129.12, 127.94, 37.12, 25.97

2. High performance liquid chromatography (HPLC) analysis

HPLC analyses were performed with a HPLC system (Agilent technologies 1260 infinity) equipped with a quaternary pump (G1311B), auto liquid sampler (G1329B), Diode array detector (G1315D) and analytical scale-fraction collector (G1364C). Instrument control, data acquisition and data analysis was performed using a ChemStation software (Agilent Technologies, Workingham, UK). A ZORBAX Eclipse plus C18 (250 x 4.6 mm) column with 5 µm particle size at room temperature was used. Mobile phase consisted of acetonitrile/water with 0.1 % TFA and the flow rate was 1.0 mL/min. Injection volume was 10 µL and the column effluent was monitored at 220 nm. Program set with the initial mobile phase composition of acetonitrile/water (10:90) with gradual increase in acetonitrile concentration from 10 % to 70 % within 20 min of run. Further acetonitrile concentration was increased from 70 % to 95 % within next 5 min of run.

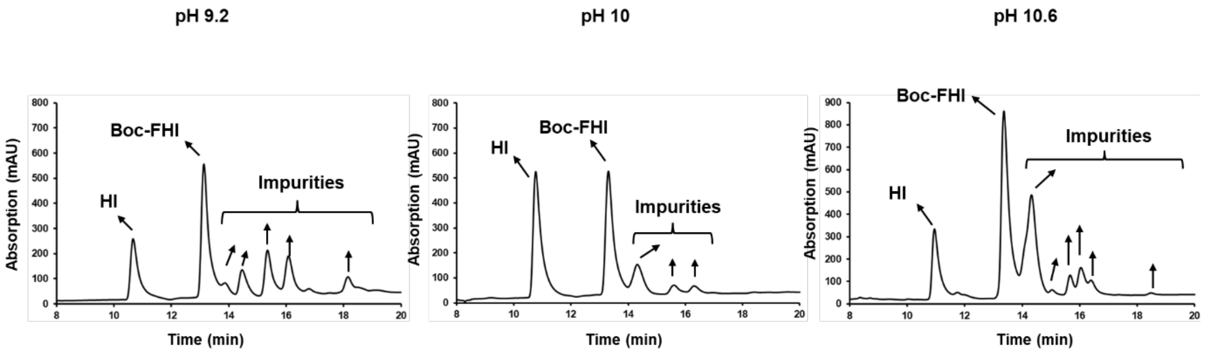

Figure S1: Analytical HPLC chromatograms of reaction mixtures (reaction between HI and Boc-Phe-OSu) after 1 h at different pHs.

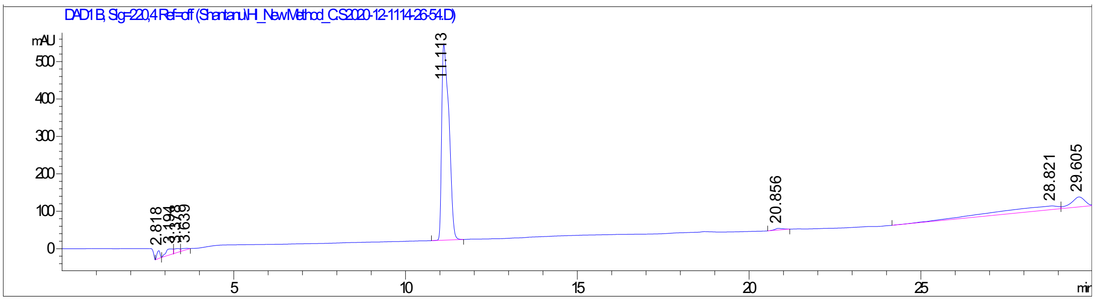

Figure S2: Analytical HPLC chromatogram of **HI** at wavelength 220 nm

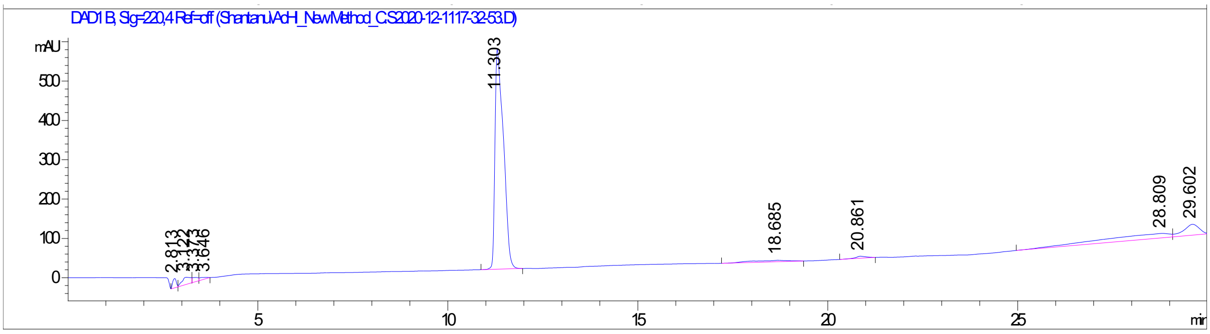

Figure S3: Analytical HPLC chromatogram of **AcHI** at wavelength 220 nm

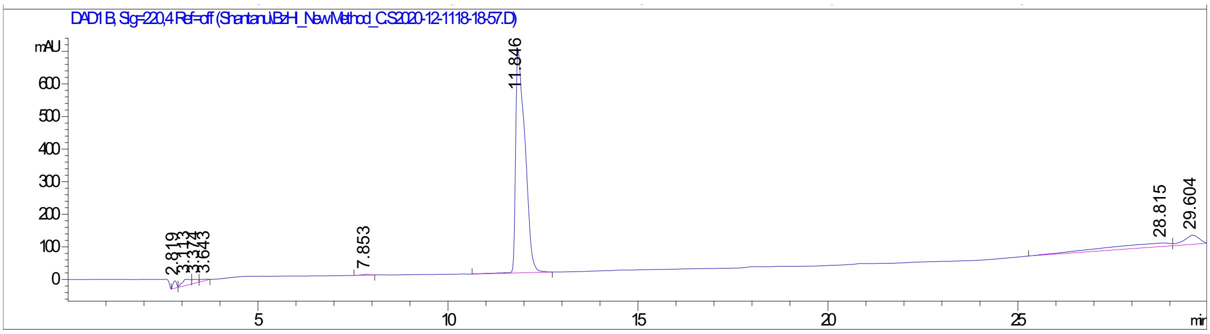

Figure S4: Analytical HPLC chromatogram of **PhacHI** at wavelength 220 nm

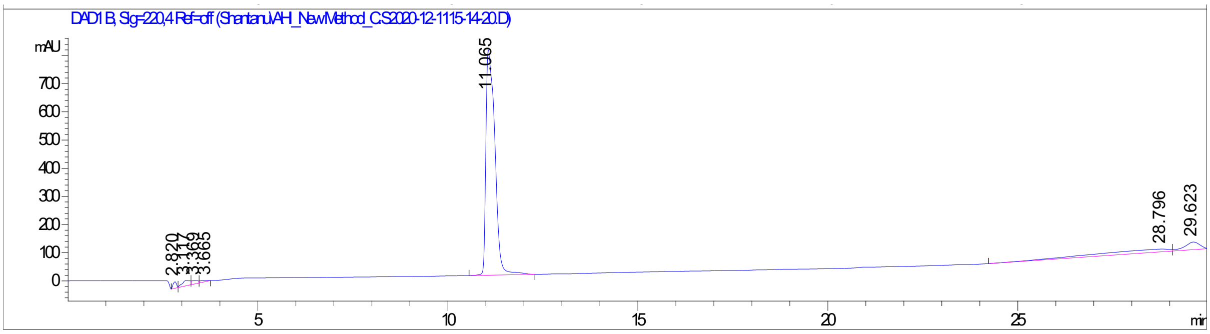

Figure S5: Analytical HPLC chromatogram of **AHI** at wavelength 220 nm

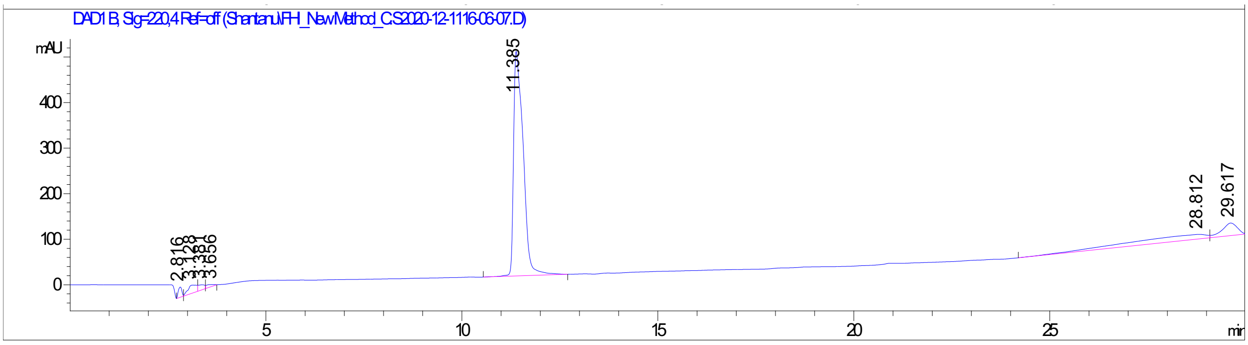

Figure S6: Analytical HPLC chromatogram of **FHI** at wavelength 220 nm

3. Mass spectrometry:

Mass spectra recorded either through electrospray ionization (ESI) or Matrix-assisted laser desorption/ionization time of flight (MALDI-ToF). For ESI-MS SCIEX X500B qToF platform paired with ExionLC AD UHPLC and Agilent 6130 mass spectrometer with Agilent 1200 series HPLC was used. Whereas MALDI-ToF data was collected with Bruker Daltonics UltrafleXtreme Software-Flex control version 3.4, using sinapic acid and α-cyano-4-hydroxycinnamic acid (HCCA) matrix and analysis was performed using Open LAB CDS A.01, SCIEX OS 1.7, Bruker Daltonics ESI Compass 1.3, and BioPharmaView Flex. Mass spectra in both cases were recorded in positive ion mode.

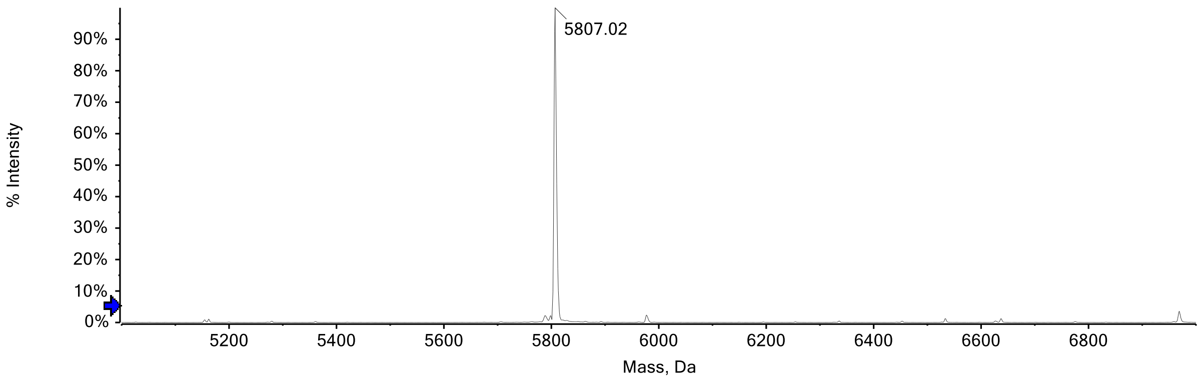

Figure S7: Mass spectrum of **HI**

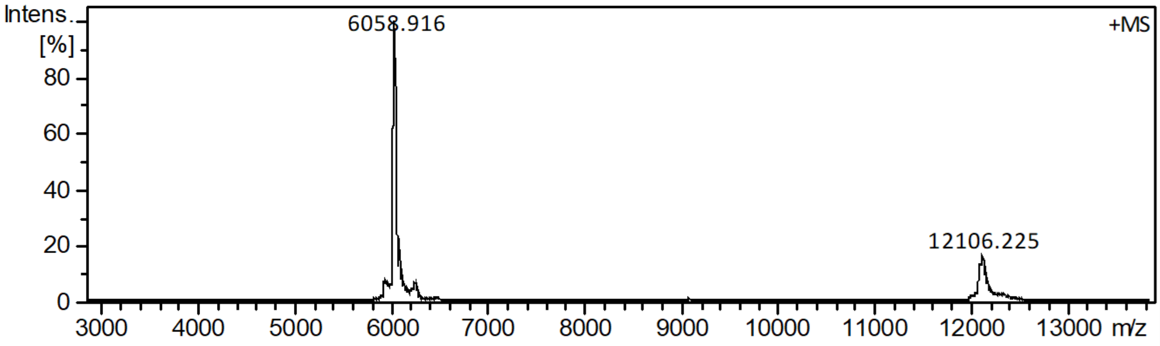

Figure S8: Mass spectrum of **BFHI**

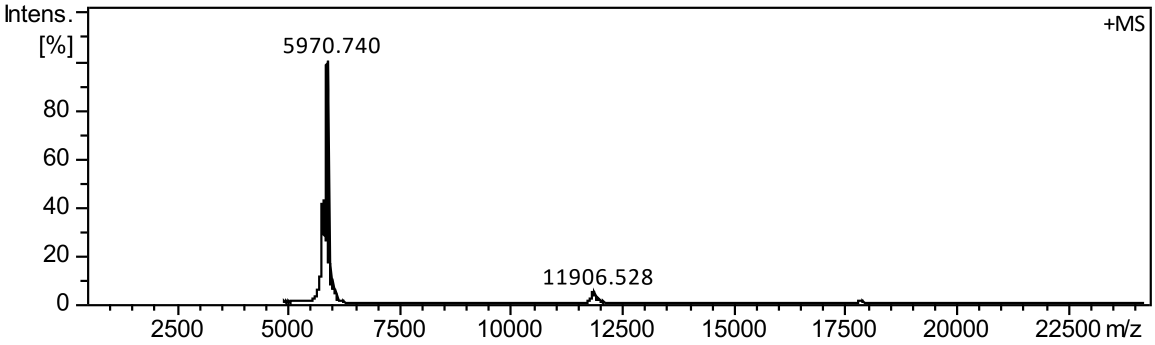

Figure S9: Mass spectrum of **BAHI**

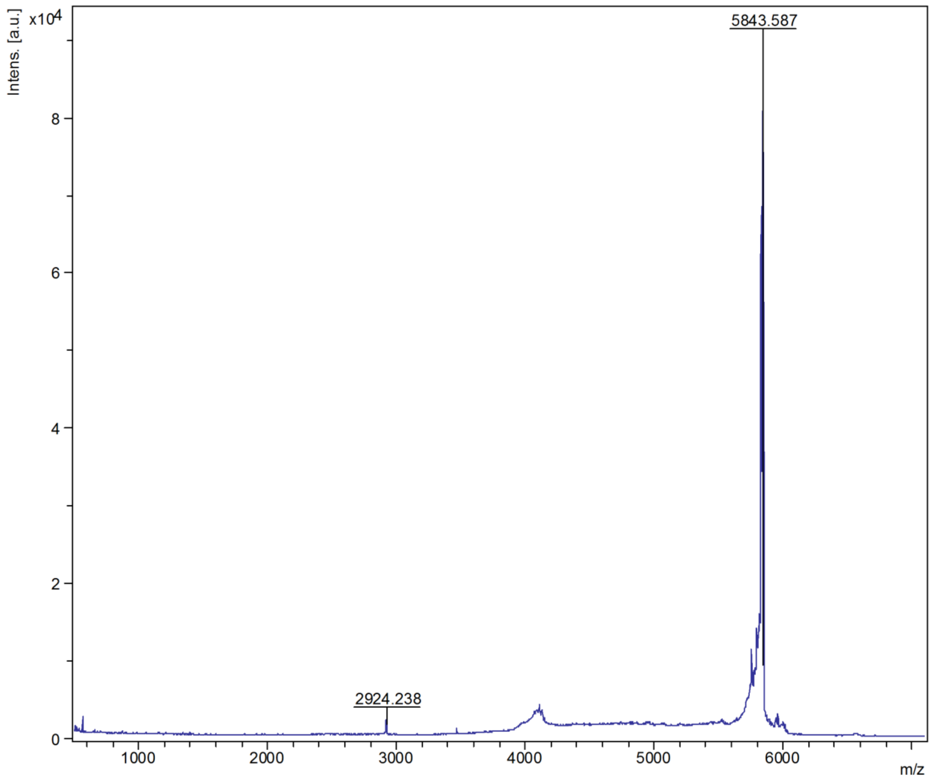

Figure S10: Mass spectrum of **AcHI**

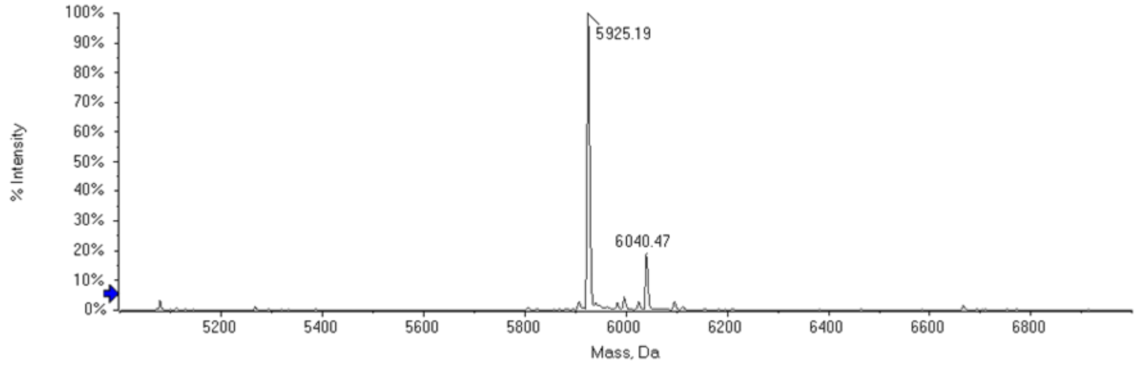

Figure S11: Mass spectrum of **PhacHI**

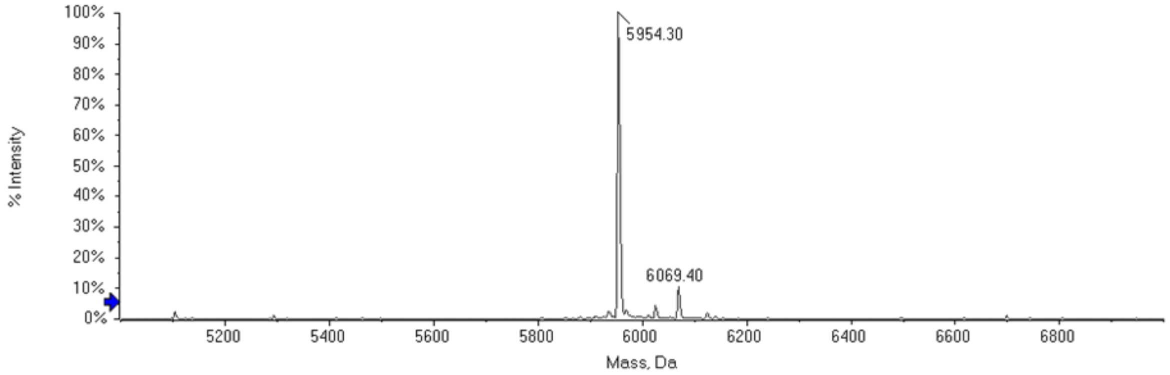

Figure S12: Mass spectrum of **FHI**

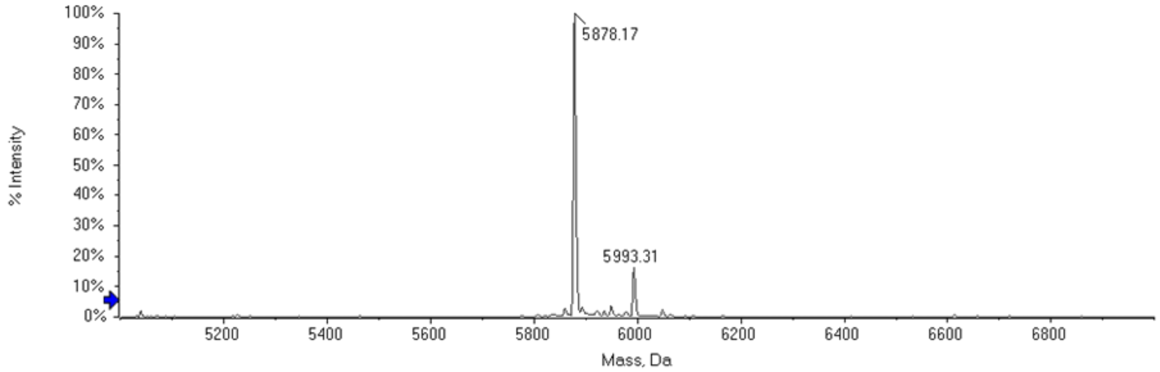

Figure S13: Mass spectrum of **AHI**

4. Copies of ^{1}H and ^{13}C NMR spectra

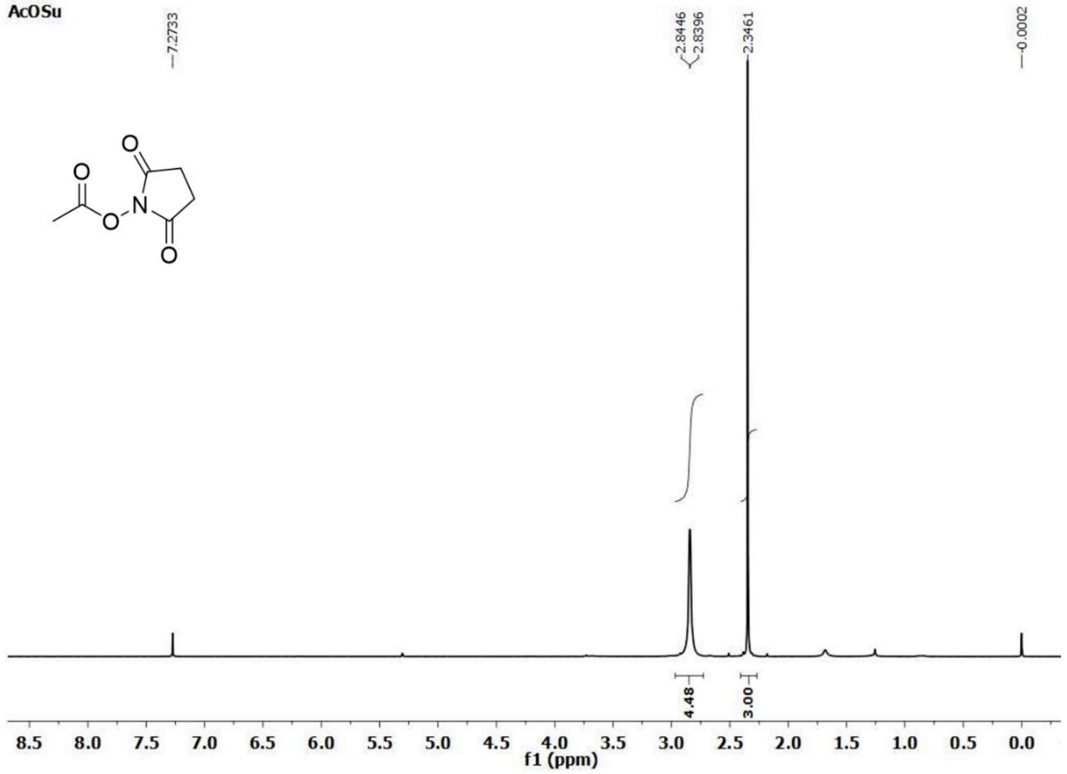

Figure S14: ^{1}H NMR spectrum of **Ac-OSu** (400 MHz, $CDCl_3$)

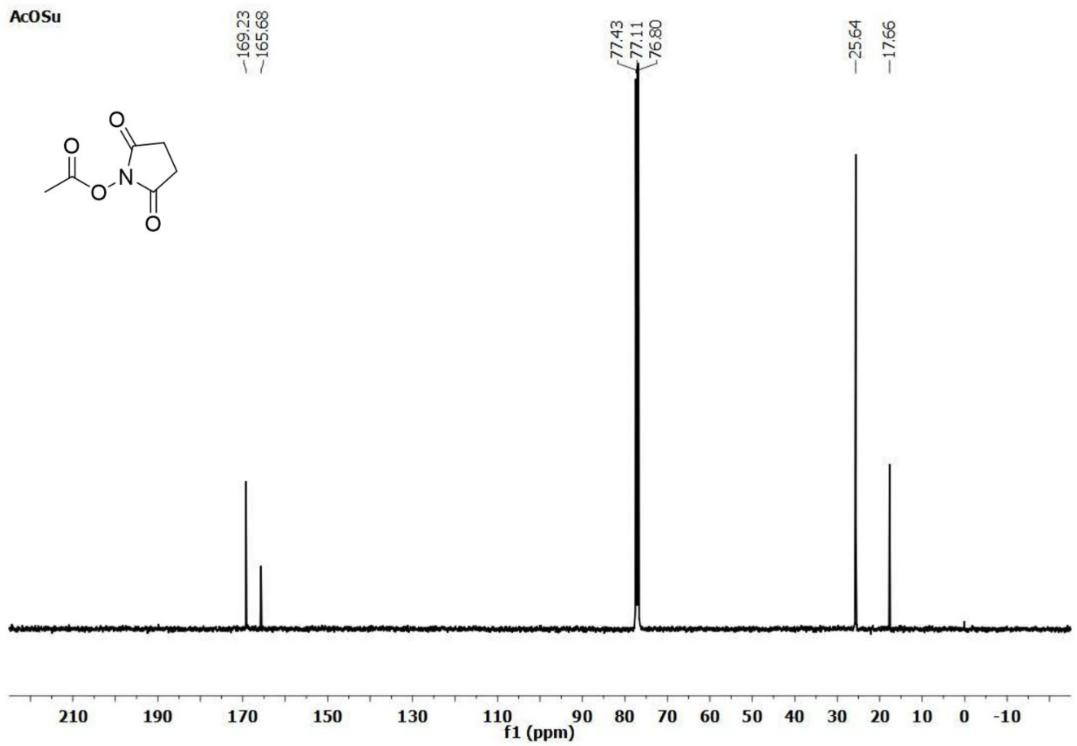

Figure S15: ^{13}C NMR spectrum of **Ac-OSu** (100 MHz, $CDCl_3$)

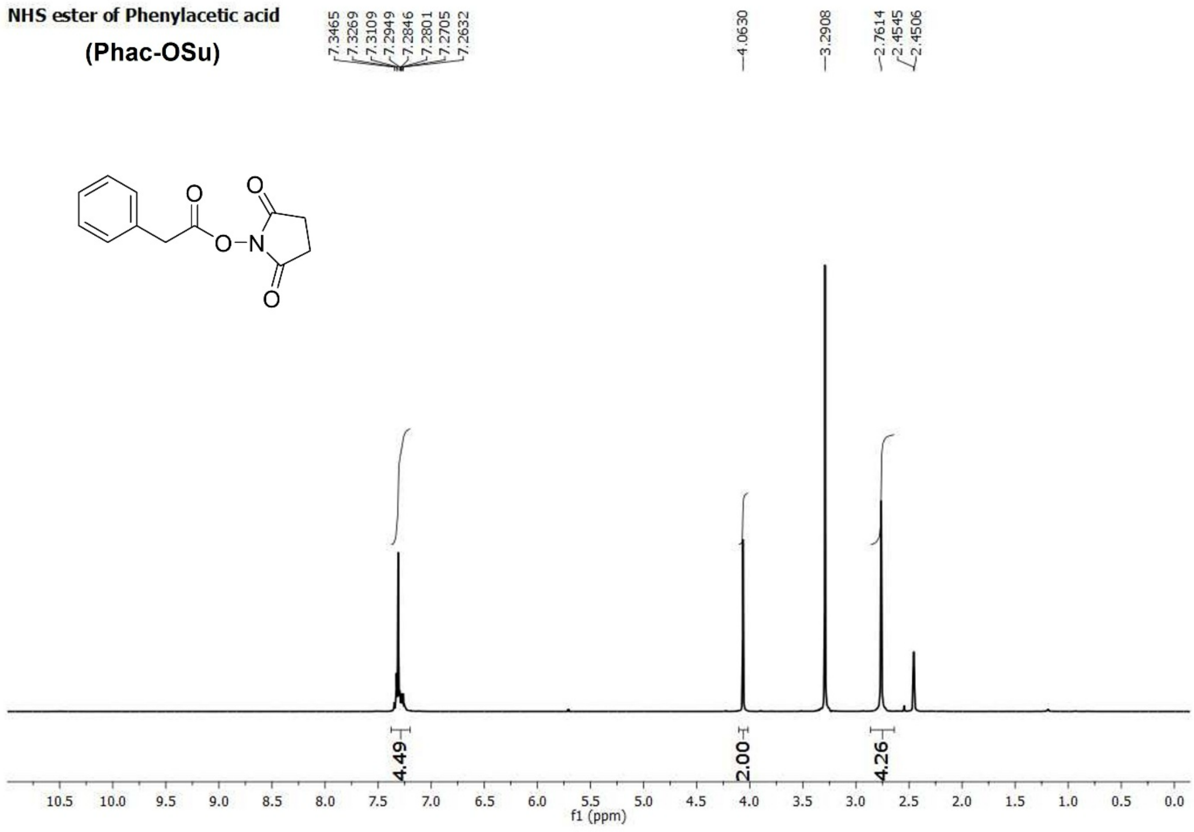

Figure S16: ^{1}H NMR spectrum of **Phac-OSu** (400 MHz, DMSO-d_6)

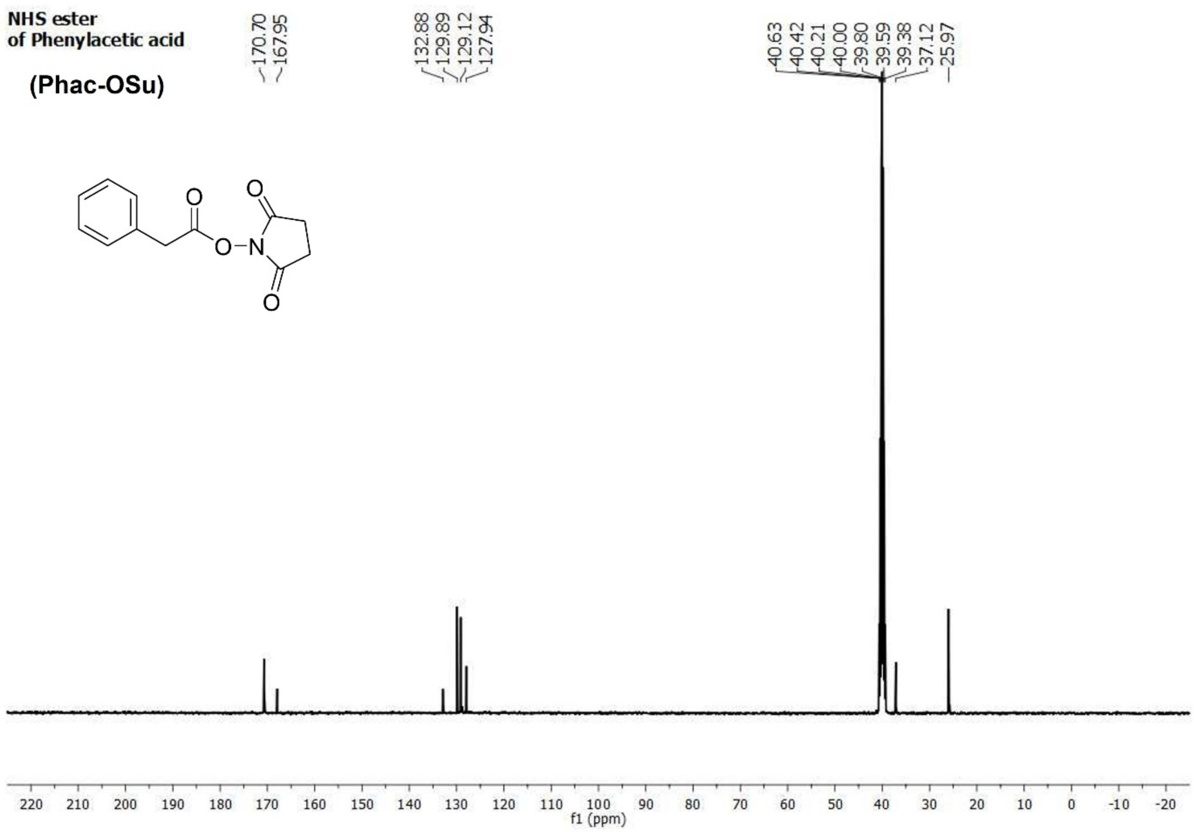

Figure S17: ^{13}C NMR spectrum of **Phac-OSu** (100 MHz, DMSO-d_6)

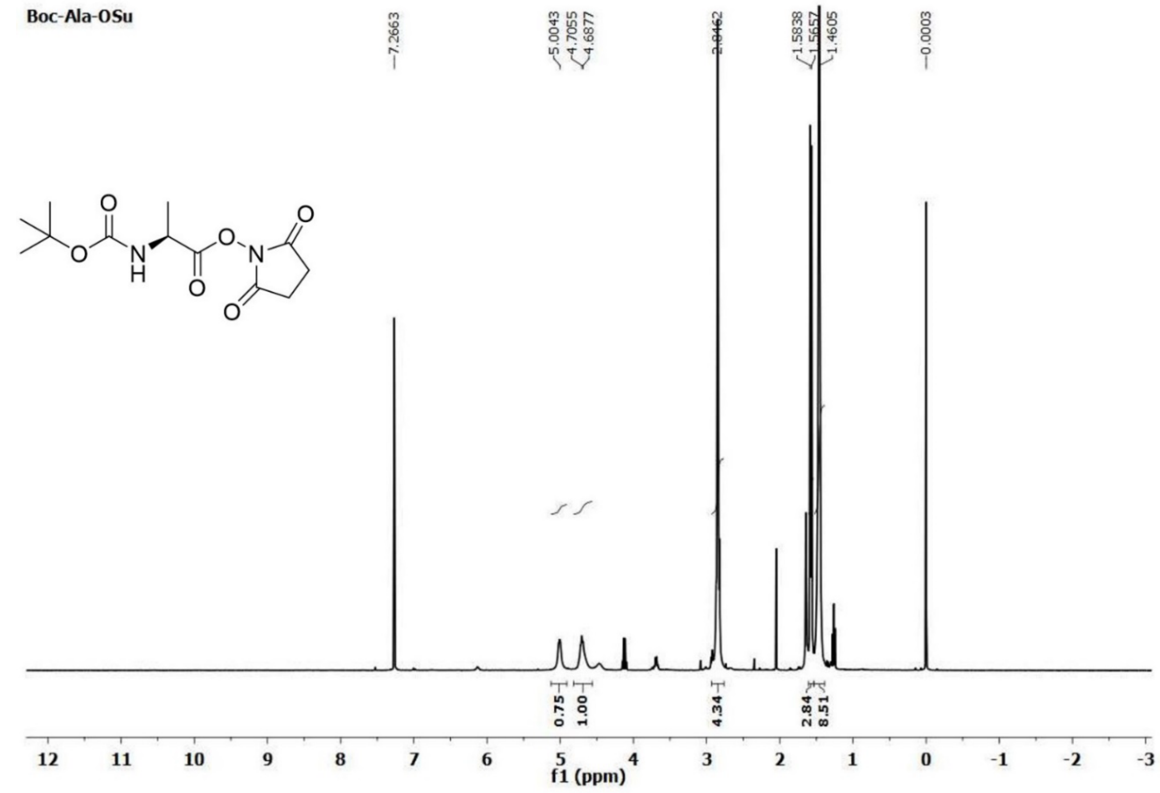

Figure S18: ^{1}H NMR spectrum of **Boc-Ala-OSu** (400 MHz, $CDCl_3$)

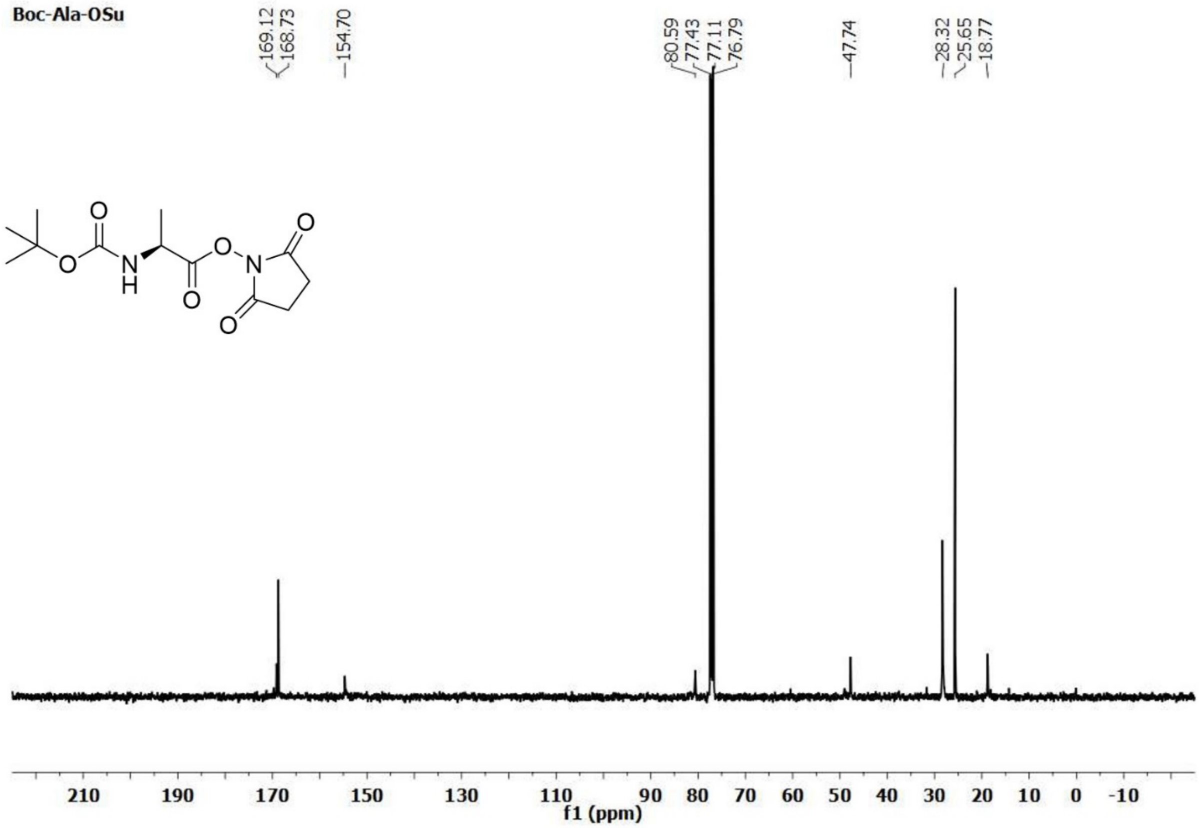

Figure S19: ^{13}C NMR spectrum of **Boc-Ala-OSu** (100 MHz, $CDCl_3$)

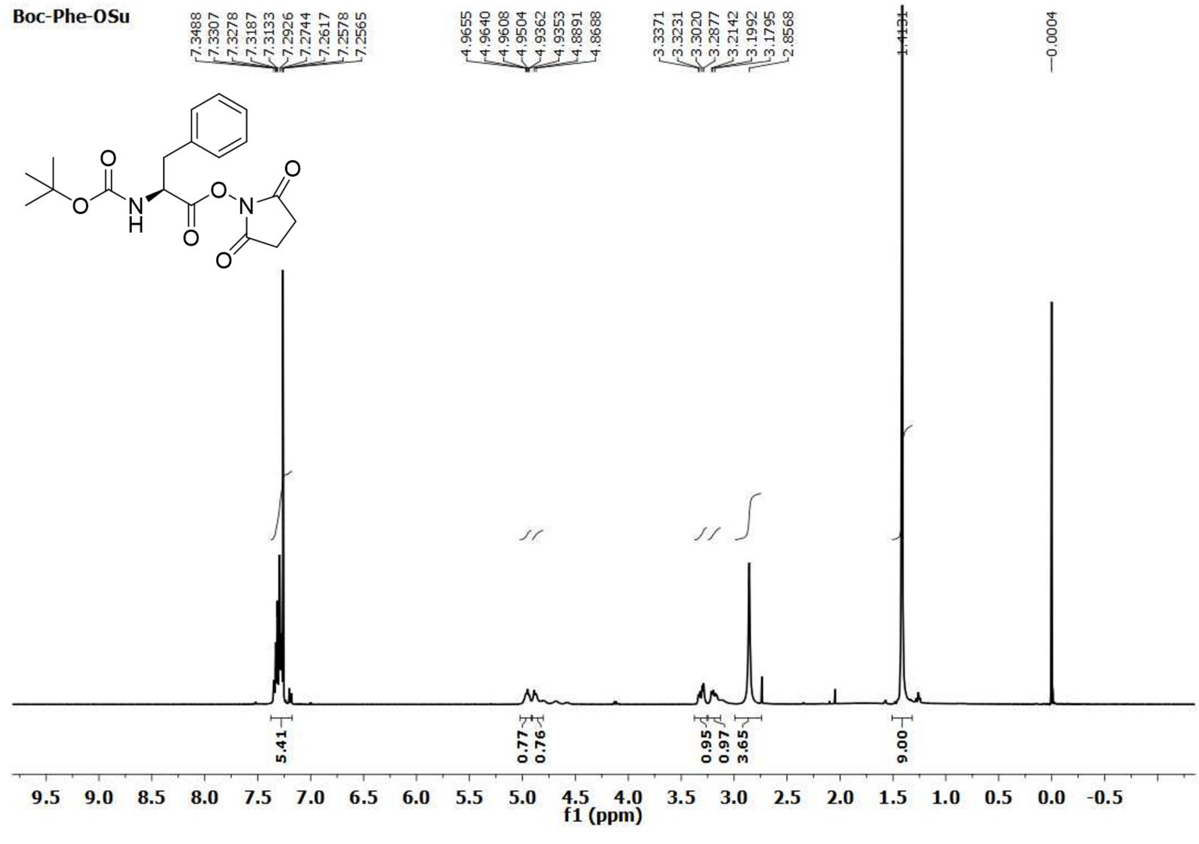

Figure S20: ^{1}H NMR spectrum of **Boc-Phe-OSu** (400 MHz, $CDCl_3$)

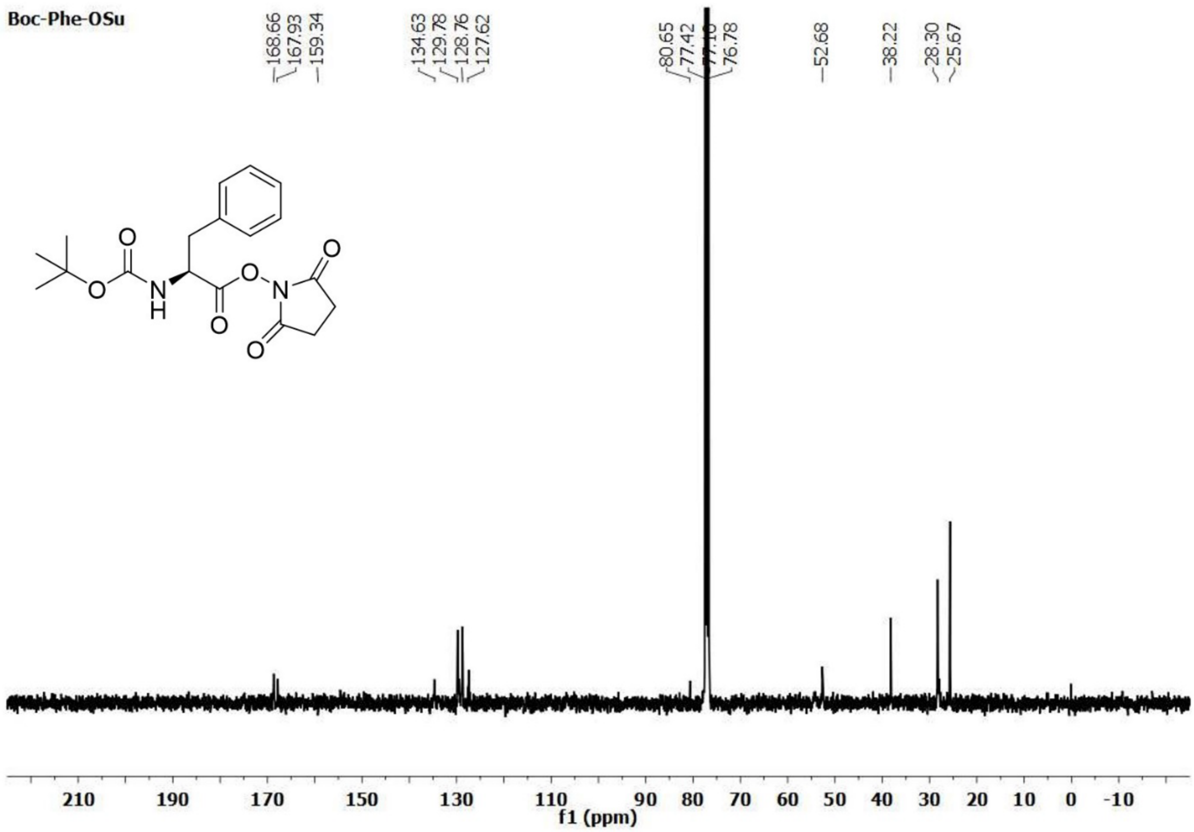

Figure S21: ^{13}C NMR spectrum of **Boc-Phe-OSu** (100 MHz, $CDCl_3$)

5. Conserved structural integrity after insulin modifications observed by Circular dichroism (CD) spectroscopy

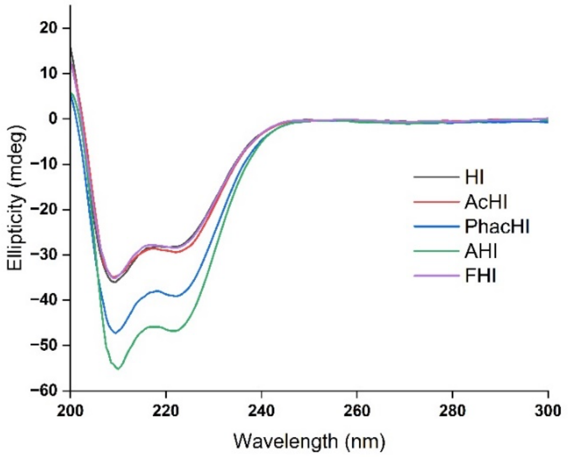

Figure S22: Circular Dichroism (CD) spectra of 0.5 mg/mL (85 μM) each of **HI**, **AcHI**, **PhacHI**, **AHI** and **FHI** in 0.1N HCl_{aq} (pH 1.6) containing 25 mM NaCl. CD experiments were carried out at 25 ± 0.1 °C using quartz cuvette with a path length of 1 mm.

6. ThT fluorescence at pH 7.4

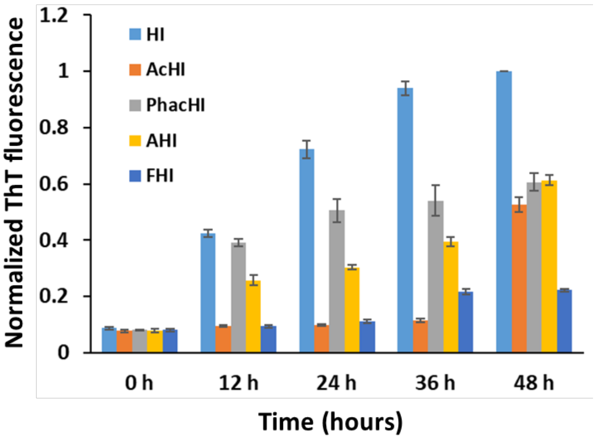

Figure S23: Normalized ThT fluorescence intensity (λ_{em}) at 488 nm of 0.5 mg/mL (85 μM) each of **HI**, **AcHI**, **PhacHI**, **AHI** and **FHI** incubated in 1X PBS buffer (pH 7.4) at 65 °C in different time intervals.

7. Insulin activity on long cold storage

Commercially available wild type insulin is known to lose its activity when stored in refrigeration for long durations, where it tends to aggregate and form toxic fibrils.[1] To test the efficacy of the modified insulin analog **FHI** as compared to that of **HI**, we stored both **HI** and **FHI** at 4 ºC for 2 months. Serum starved HEK293T cells were then treated with both **HI** and **FHI** at a concentration of 0.5 μmol for 30 minutes and the level of phosphorylated AKT was measured using western blot analysis for specific antibody as a read out of insulin activity. Interestingly, the modified insulin **FHI** retained its activity in the present treatment conditions as compared to the untreated controls whereas the human recombinant insulin **HI** failed to show any significant difference with the untreated control in Akt phosphorylation suggesting that **FHI** is far more stable than the wild type insulin.

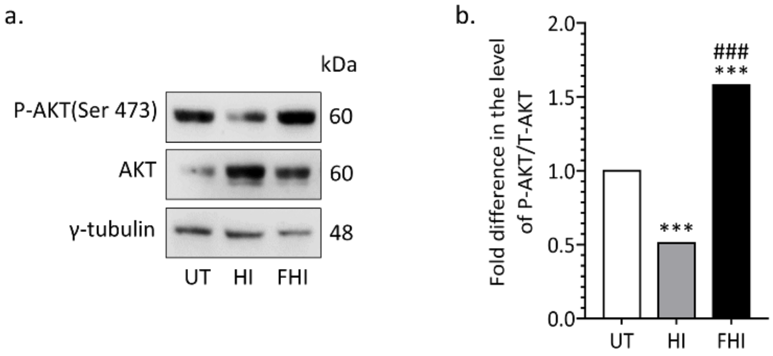

Figure S24: Comparative *in-vitro* efficacy of FHI in HEK293T cell line upon refrigeration at 4 °C for 2 months. a. Representative immunoblots showing the relative levels of P-AKT (Ser 473) in the

HEK293T cell lines that are untreated **(UT)** or treated for 30 min with 0.5 µm of Human insulin **(HI)** or the modified phenylalanine conjugated human insulin **(FHI)** that was stored at 4℃ for 2 months. γ-tubulin served as the loading control. **b.** Bar diagram showing the fold change in intensities (measured by densiometric analysis) of P-AKT normalised to T-AKT levels in **HI** or **FHI** treated HEK293T cells as compared to the **UT** control**. (**N=3; Ordinary One-way ANOVA analysis; ****p*≤0.001 when compared to **UT** control and ###*p*≤0.001 when compared to **HI**, Error bars represent S.E of mean).

8. Molecular Dynamics Simulations

Initial coordinates for the MD simulations were taken from the NMR structure of the human insulin monomer in 35% CD_3CN zinc free PDB: 2JV1.[2] The protonation state of residues was determined by H++ server[3] corresponding to pH of 1.6. It is known that under these acidic pH, HI is in monomeric form.[4] MD simulations were performed with AMBER-18 PMEMD software.[5,6,7,8] AMBER ff14SB force-field[9] and TIP3P water model[10] were used. The **HI** (**FHI**) monomer was solvated in a 53 x 59 x 53 $Å^3$ (53 x 66 x 53 $Å^3$) box having 3789 (4233) water molecules. First, 30000 steps of steepest-descent minimization was performed and then equilibrated at 338 K (65 ℃) for 2.2 ns in NPT ensemble. After a 20 ns long NVT ensemble equilibration, we performed 1 µs NVT production run. Berendsen barostat[11] is used to maintain the pressure and Langevin thermostat is used to maintain the temperature with collision frequency 1 ps^{-1}. Bonds involving hydrogens were constrained using SHAKE algorithm[12] and a time step of 1 fs is used.

Force Field Parameterization. The B29Lys(Phe) residue was prepared using conventional AMBER protocols and was capped to generate the correct force-field parameters. The structure was then optimized using Avogadro.[13,14] The AmberMD tool package Antechamber 19.0[15] was used to generate GAFF force-fields and AM1-BCC charges.

Analysis of Molecular Dynamics Trajectories. The analysis of MD trajectories was done by MDTraj software package.[16]

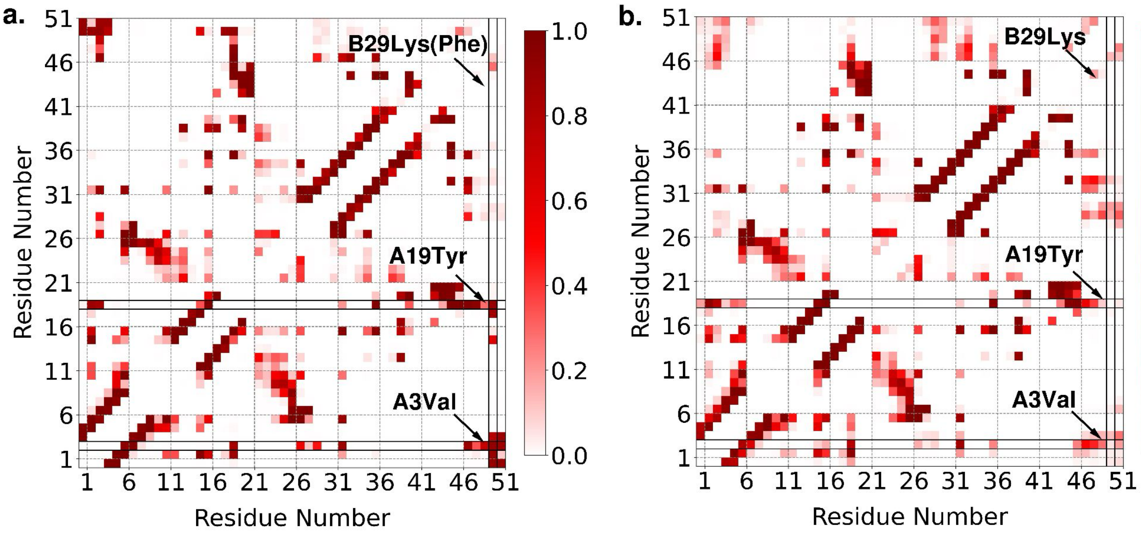

Figure S25. Contact map of **a. FHI** and **b. HI.** The color bar denotes the fraction of time when the contact is present between two residues.

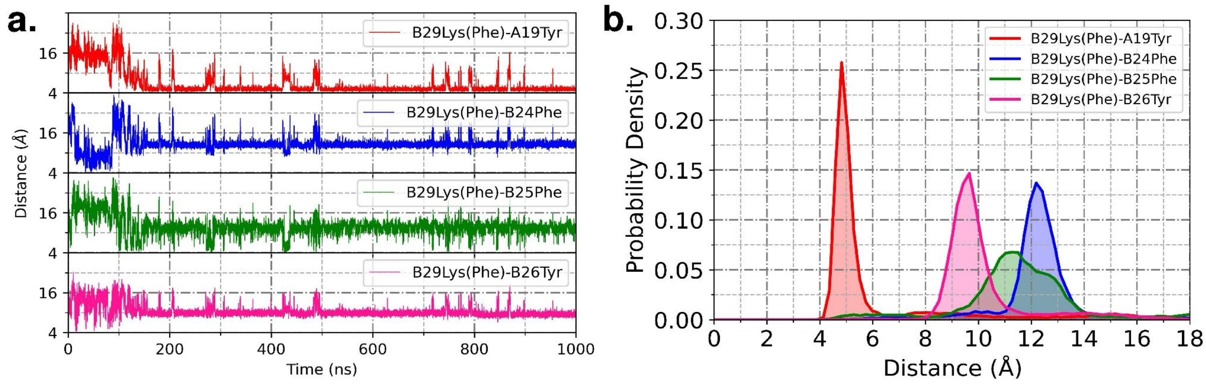

Figure S26. Aromatic interactions in **FHI** between B29Lys(Phe) and other aromatic residues. **a.** Distance between COM of phenyl rings of B29Lys(Phe) and A19Tyr, B24Phe, B25Phe, and B26Tyr with time, and **b.** their probability distribution.

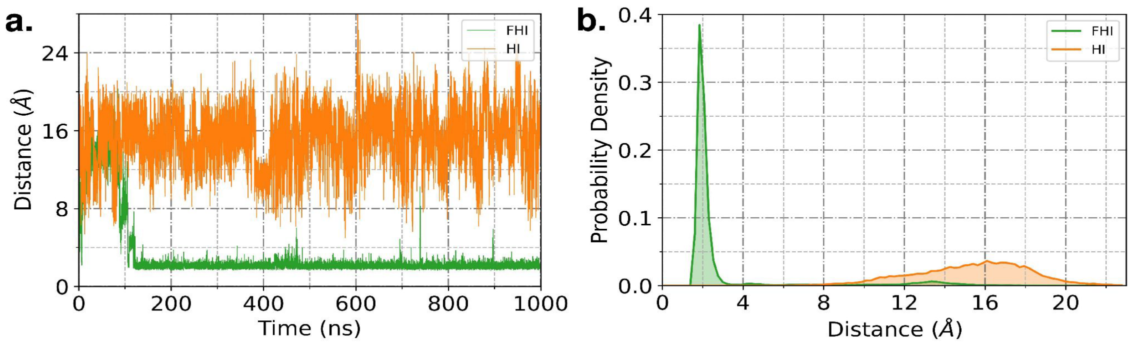

Figure S27. Hydrogen bonding interactions between B29 and A3 residues. **a.** Distance between backbone carbonyl oxygen of B29Lys(Phe)/B29Lys and backbone amide hydrogen of A3Val in **FHI** and **HI** residues with time, and **b.** their probability distribution.

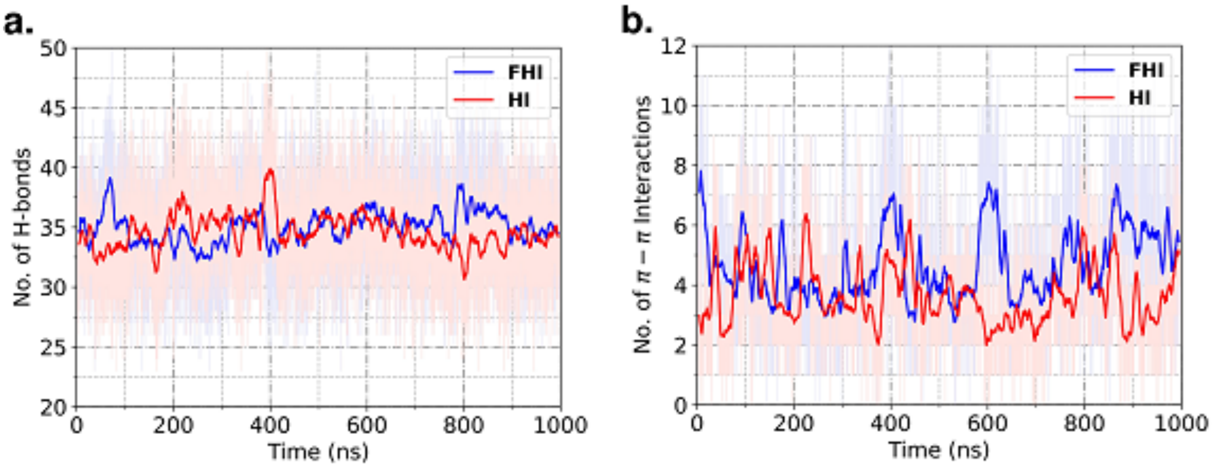

Figure S28. Total no. of **a.** H-bonds and **b.** π-π aromatic interactions in HI and FHI protein with time. Solid-thick lines indicate the block average of every 10 ns data, while the thin lines indicate the instantaneous values.

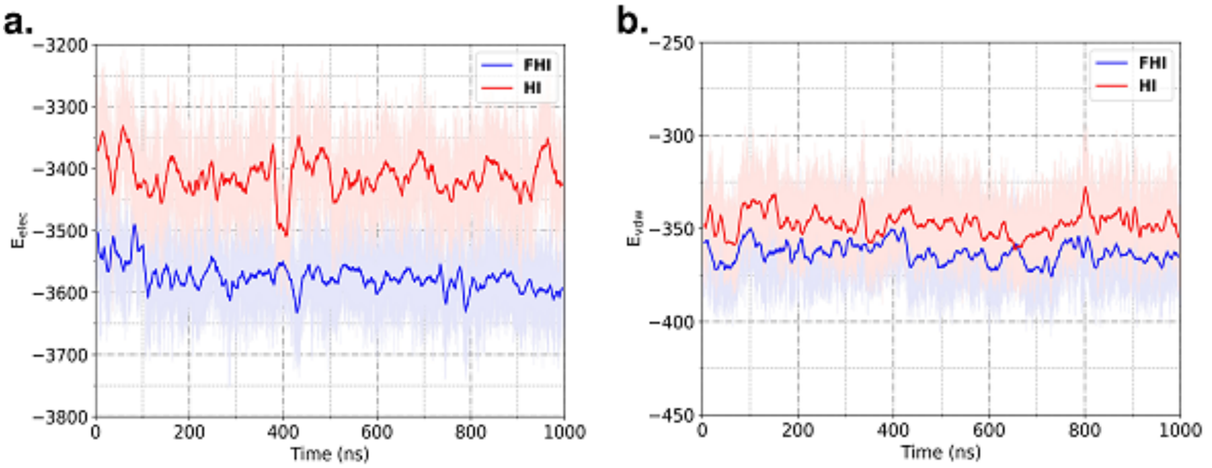

Figure S29. a. Electrostatic energy (E_{elec}) and **b.** van der Waals (E_{vdw}) energy in HI and FHI protein with time. Solid-thick lines indicate the block average for every 10 ns data, while the thin lines indicate the instantaneous values.

9. Oligomerization status of HI and FHI

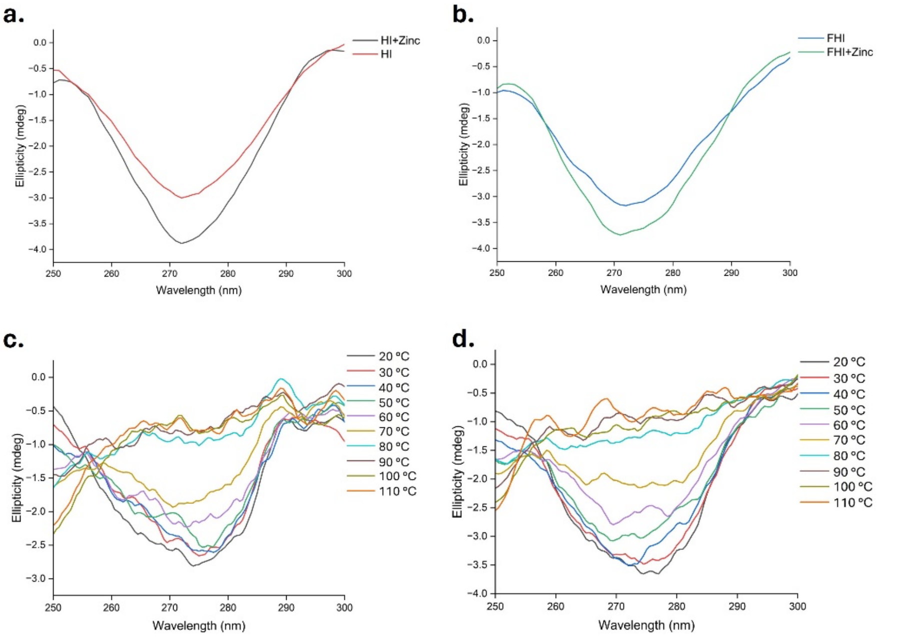

Figure S30. CD spectra of a. HI (0.3 mM) solution in absence or presence of zinc (0.3 mM). b. FHI (0.3 mM) solution in absence or presence of zinc (0.3 mM). c. HI (0.3 mM) solution in presence of zinc (0.3 mM) scanned under temperature gradient of 20 °C-110 °C (Temperature gradient 2°C/min). d. FHI (0.3 mM) solution in presence of zinc (0.3 mM) scanned under temperature gradient of 20 °C-110 °C (Temperature gradient 2°C/min).

10. Melting temperature of HI and FHI

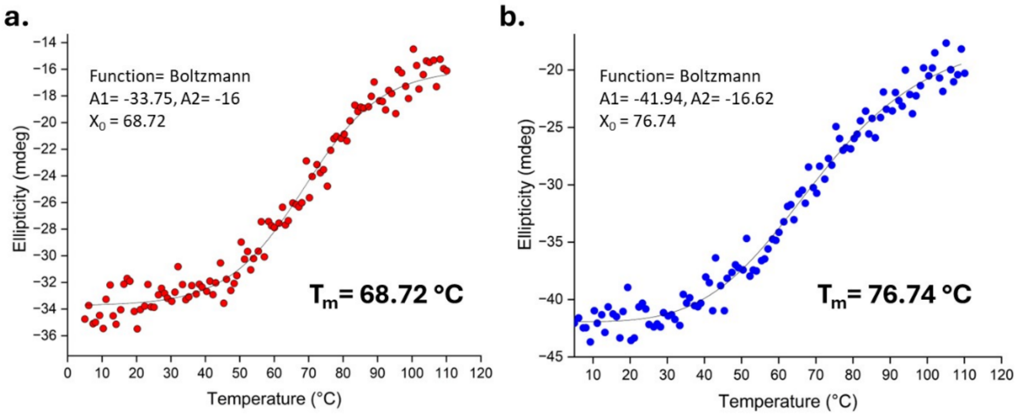

Figure S31. Melting temperature (T_m) is calculated for both HI and FHI by plotting their respective ellipticity at 222 nm against their corresponding temperatures. T_m is calculated by fitting the values in Boltzmann sigmoid equation. Circular Dichroism (CD) spectra were taken at concentration of 0.5 mg/mL (85 µM) each of **HI** and **FHI** in 0.1N HCl_{aq} (pH 1.6) containing 25 mM NaCl. Experiments were carried out at 25 ± 0.1 °C using quartz cuvette with a path length of 1 mm.

11. HPLC analyses of the samples after heat treatment

To quantitatively evaluate the soluble populations of both HI and FHI after heat treatment, we performed HPLC analysis in Agilent 1260 infinity machine using Poroshell-120 EC-C18 (150 × 3 mm, 2.7 µm) reverse phase column. We plotted the area under the curve (AUC) of their corresponding peaks in the HPLC chromatograms for both fresh and heated (65 °C) samples at acidic (pH 1.6, Fig. S32) and physiological (pH 7.4, Fig. S33) pH levels.

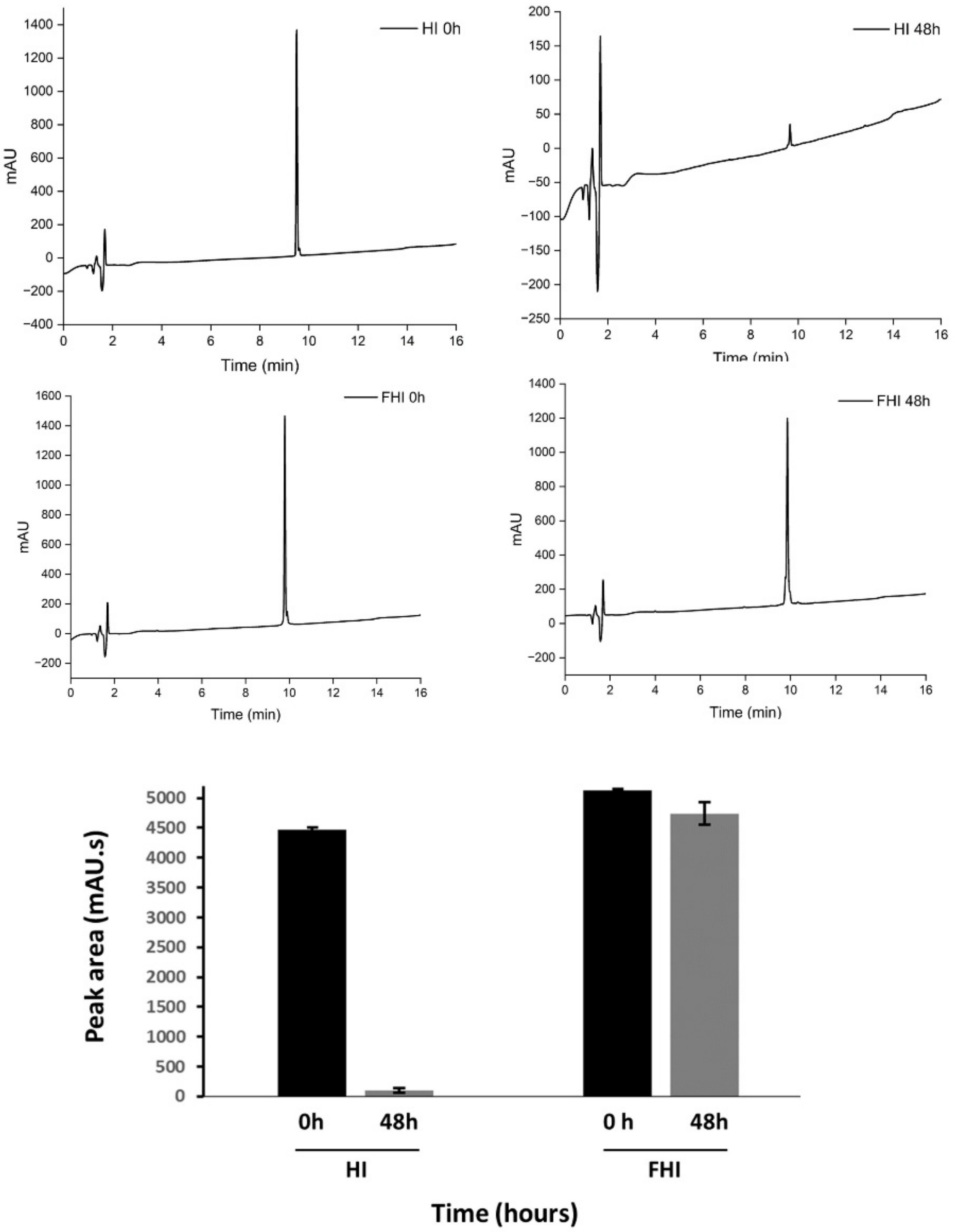

Figure S32. HPLC analysis and quantification of fresh as well as heated (65 °C) samples of HI and FHI at pH 1.6.

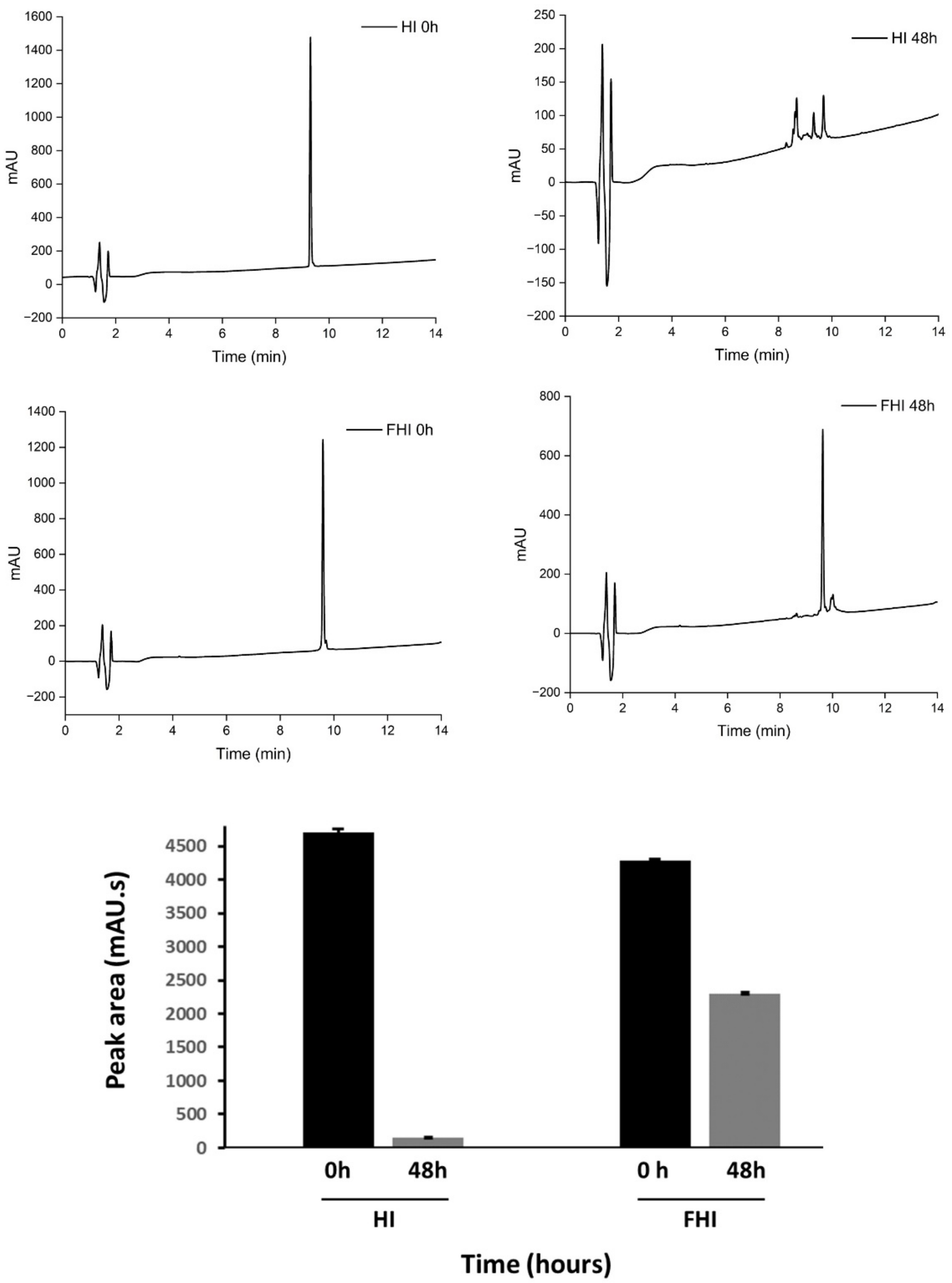

Figure S33. HPLC analysis and quantification of fresh as well as heated (65 °C) samples of HI and FHI at pH 7.4.

12. Mass analyses of the samples after heat treatment

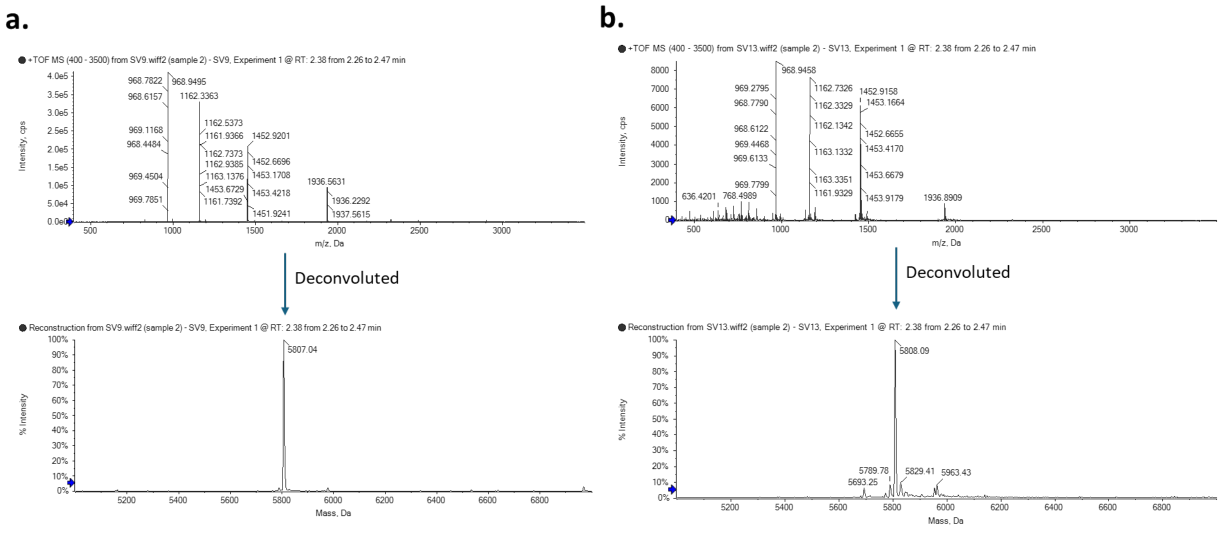

Figure S34. Mass spectra of HI samples incubated at pH 1.6 (0.1 N HCl, 25 mM NaCl) for **a.** 0h, **b.** 48h.

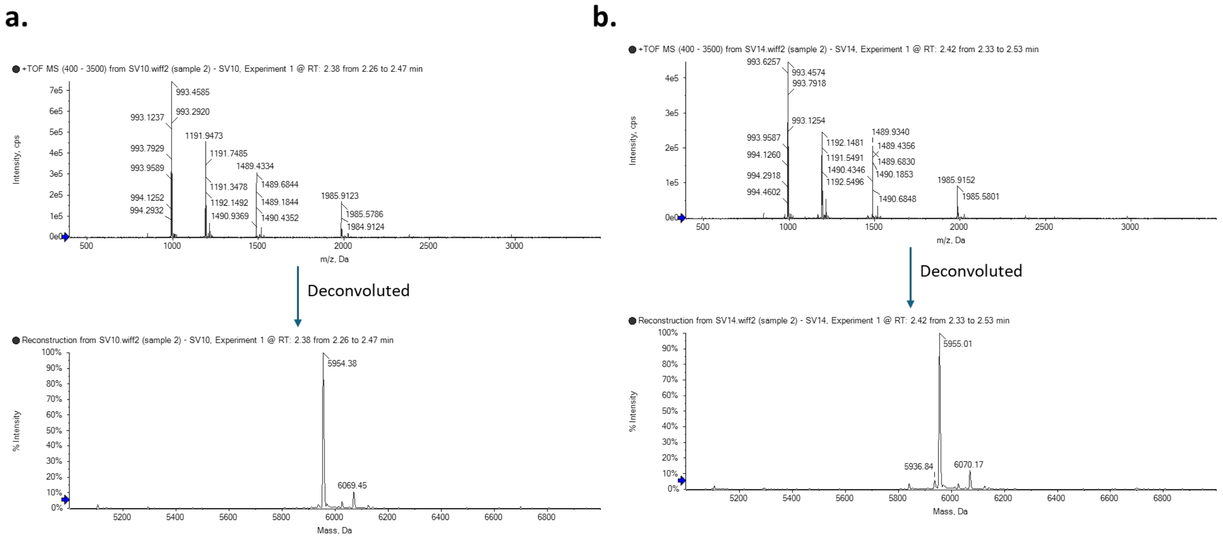

Figure S35. Mass spectra of FHI samples incubated at pH 1.6 (0.1 N HCl, 25 mM NaCl) for **a.** 0h, **b.** 48h.

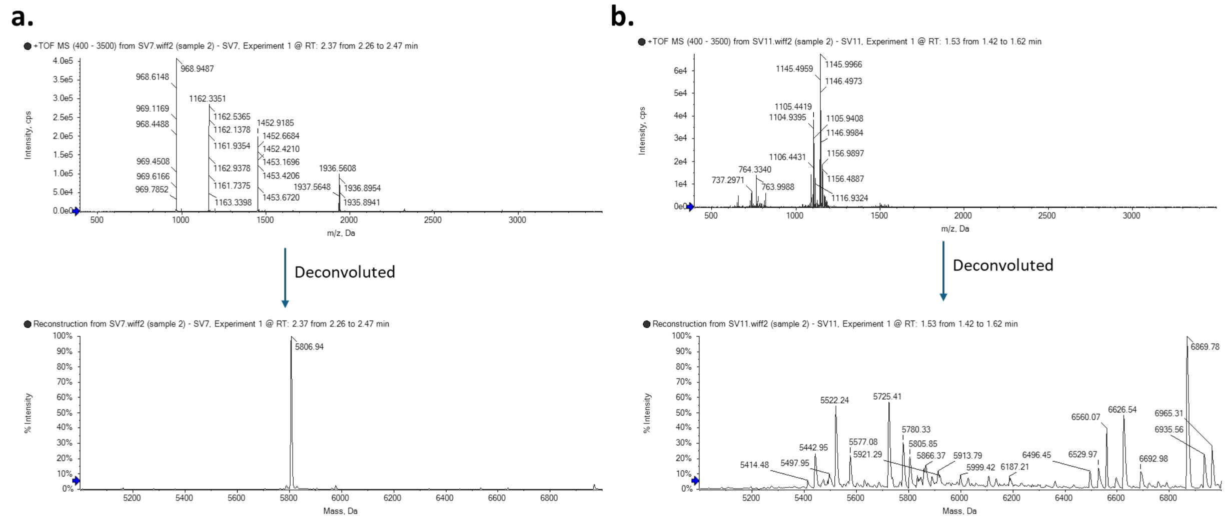

Figure S36. Mass spectra of HI samples incubated at pH 7.4 for **a.** 0h, **b.** 48h.

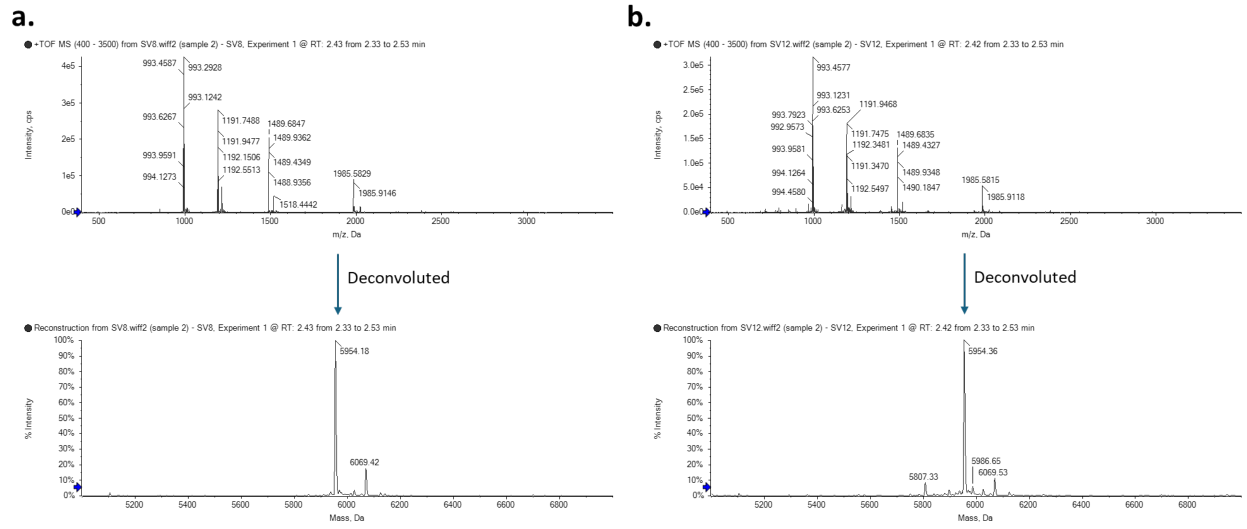

Figure S37. Mass spectra of FHI samples incubated at pH 7.4 for **a.** 0h, **b.** 48h.

13. B29 Lys modifications in insulin

Table S1. B29 Lys modifications in insulin

Existing studies	Name	Added moieties	Purpose
	Insulin degludec	Hexadecanedioic acid	Long-acting
	Insulin detemir	Myristic acid	Long-acting
	Insulin-327	A fatty diacid	Hepatoselective insulin
	PBA-insulin	Phenyl boronic acid (PBA)	Glucose-responsive insulin
	W99-S-32	Palmitic acid	Long-acting
**Current study**	AcHI	Acetyl	Enhancing thermostability
	PhacHI	Phenylacetyl	
	AHI	Alanyl	
	FHI	Phenylalanyl	

14. Comparison of current work with previous study

Table S2. Comparison of the current study with the insulin analog reported in *Protein J.*, **2023**, *42*, 383.

Sl. No.	Experiments & Observations	Insulin analog reported in *Protein J.*, 2023, *42*, 383	Current Study	Remarks: advantages in the present study
1	Purification of modified insulin	No HPLC purification to isolate modified insulin	HPLC purification was done	Purity of newly synthesized analogs were assessed through analytical HPLC in this work.
2	Specific modification at B29 position	Tri-acetylated at positions Gly A1, Phe B1 and Lys B29 positions, but acetylation not carried out specifically at B29 Lys position	Single modification: specifically at Lys B29 position	Different single modifications have been introduced with diverse molecular structures at B29 ε-amine.

3	Mass analysis of modified insulin	Not reported	Reported	ESI and MALDI mass analyses have been reported in this work to confirm modifications and purity
4	Chemical Modification specificity	Not reported	Through mass analysis	Specific chemical conjugation has been assessed through Mass and HPLC in this work.
5	Melting temperature	Native insulin (66.5 °C), Modified insulin (72.5 °C)	Native insulin (68.7 °C), Modified insulin, FHI (76.7 °C)	More gap in melting temperature has been achieved in this work.
6	Experimental pH	pH 5 and 7.4	pH 1.6 and 7.4	Acidic pH was used in this study for more rigorous amyloidogenic screening conditions to deduce comparative thermostability
7	Incubation conditions	No salt stress during incubation	25 mM NaCl	Salt stress provide more stringent amyloidogenic environment in this study
		37 °C & 50 °C	65 °C	All the studies were carried out at higher temperature in this study: 15 °C higher than corresponding comparision
		Prolonged heat treatment has not been carried out	Prolonged heat treatment till 48 hours has been carried out	Prolonged heat treatment and their time-dependent thermostability status has been reported in this study
8	Bioactivity after chemical modification and heat treatment	Bioactivity has not been reported	Bioactivity in *in vitro*, *ex vivo* and *in vivo* conditions were analysed	Modified insulins are bioactive not only in fresh condition, but also after heat-treatment for prolonged duration
9	Time-dependent assay	Not reported	Reported till 48 hours	Time-dependent aggregation kinetics and their bioactivity status have been reported in this study
10	Molecular dynamics simulations study	Not reported	Reported	Enhanced thermostability correlated with additional favourable interactions observed during simulations trajectory in this study

15. Uncropped immunoblots

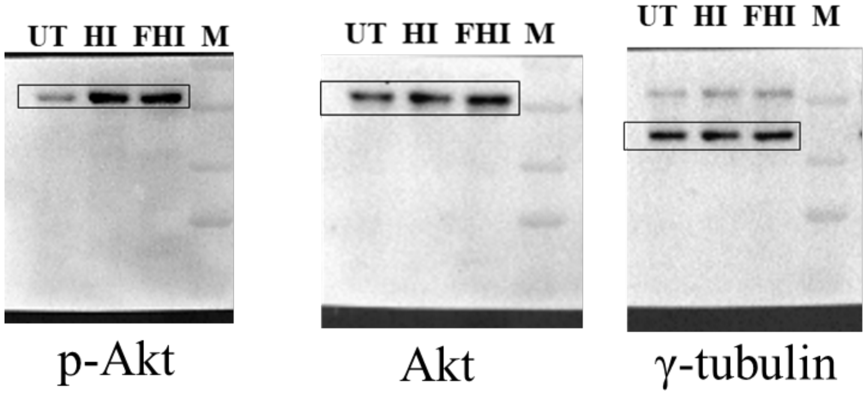

Figure S38. Uncropped immunoblots of figure 3a (M represents molecular weight marker).

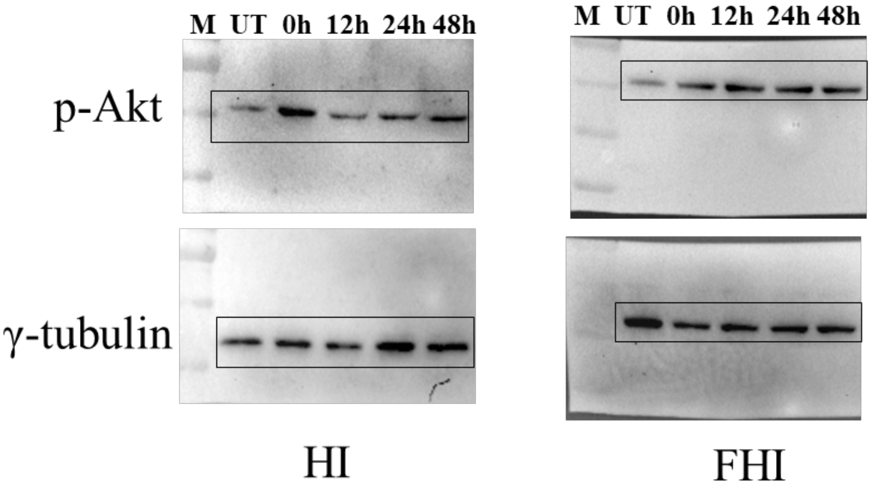

Figure S39. Uncropped immunoblots of figure 4a (M represents molecular weight marker).

16. Supplementary references

1. Heinemann, L., Braune, K., Carter, A., Zayani, A., Krämer, L.A. Insulin Storage: A Critical Reappraisal. *Journal of Diabetes Science and Technology* **15**, 147-159 (2021).
2. Bocian, W., Sitkowski, J., Bednarek, E. *et al.* Structure of human insulin monomer in water/acetonitrile solution. *J. Biomol. NMR* **40**, 55–64 (2008).
3. Anandakrishnan, R., Aguilar, B., Onufriev, A. V. *H*++ 3.0: automating p*K* prediction and the preparation of biomolecular structures for atomistic molecular modeling and simulations, *Nucleic Acids Res.*, **40**, 537–541 (2012).
4. Choi, J.H., May, B.C., Wille, H. Cohen, F.E. Molecular modeling of the misfolded insulin subunit and amyloid fibril. Biophys J. **97**, 3187-95 (2009).
5. Case, D. A. et al. AMBER 18 ; University of California, San Francisco, 2018.
6. Götz, A. W., Williamson, M.J., Xu, D., Poole, D., Le Grand, S., Walker, R. C. Routine Microsecond Molecular Dynamics Simulations with AMBER on GPUs. 1. Generalized Born. *J. Chem. Theory Comput.* **8**, 5, 1542–1555 (2012).
7. Salomon-Ferrer, R., Götz, A. W., Poole, D., Le Grand, S., Walker, R. C. Routine Microsecond Molecular Dynamics Simulations with AMBER on GPUs. 2. Explicit Solvent Particle Mesh Ewald. *J. Chem. Theory Comput.* **9**, 3878–3888 (2013).
8. Le Grand, S., Götz, A. W., and Walker, R. C., SPFP: Speed without compromise—A mixed precision model for GPU accelerated molecular dynamics simulations, *Comput. Phys. Commun*. **184**, no. 2, 374–380, (2013).
9. Maier, J. A., Martinez, C., Kasavajhala, K., Wickstrom, L., Hauser, K. E., and Simmerling, C. ff14SB: Improving the Accuracy of Protein Side Chain and Backbone Parameters from ff99SB *J. Chem. Theory Comput.* ***11*** (8), 3696-3713 (2015).
10. Jorgensen, W. L., Chandrasekhar, J., Madura, J. D., Impey, R. W., Klein, M. L. Comparison of simple potential functions for simulating liquid water. J. Chem. Phys. ***79***, 926–935 (1983).
11. Berendsen*,* H. J. C., Postma*,* J. P. M., van Gunsteren*,* W. F., DiNola*,* A. *and* Haak, J. R. Molecular dynamics with coupling to an external bath, *J. Chem. Phys.* **81**, 3684-3690 (1984)
12. Ryckaert, J.-P., Ciccotti, G., Berendsen, H. J. Numerical integration of the cartesian equations of motion of a system with constraints: molecular dynamics of n-alkanes. *J. Comput. Phys.* **23**, 327–341 (1977).
13. Avogadro: an open-source molecular builder and visualization tool. Version 1.1.1. http://avogadro.cc/
14. Hanwell, M. D., Curtis, D. E., Lonie, D. C., Vandermeersch, T., Zurek, E. and Hutchison E. Avogadro: An advanced semantic chemical editor, visualization, and analysis platform *J. Cheminformatics* **4**,17 (2012).
15. Wang J., Wang W., Kollman P. A., Case D. A. Automatic atom type and bond type perception in molecular mechanical calculations. *J. Mol. Graph. Model.* **25**, 247-60 (2006).
16. McGibbon, R. T. et al. MDTraj: A Modern Open Library for the Analysis of Molecular Dynamics Trajectories. *Biophys. J.* **109**, 1528-1532 (2015).
