# Supplementary material for: Synthesis of a highly thermostable insulin by phenylalanine conjugation at B29 Lysine
Source: Commun Chem. 2024 Jul 23;7:161. doi: 10.1038/s42004-024-01241-z (PMC11266353; doi:10.1038/s42004-024-01241-z)
Supplement: Supplementary file 2 — Description of Additional Supplementary Files [file 42004_2024_1241_MOESM2_ESM.pdf]

# Description of Additional Supplementary Files

**File name: Supplementary Data 1**

**Description**: Contains excel file having numerical source data for graphs

**File name: Supplementary Data 2**

**Description**: PDB file having initial MD  simulation structure of HI

**File name: Supplementary Data 3**

**Description**: PDB file having final MD  simulation structure of HI

**File name: Supplementary Data 4**

**Description**: PDB file having initial MD  simulation structure of FHI

**File name: Supplementary Data 5**

**Description**: PDB file having final MD  simulation structure of FHI
